# Effective Concrete Failure Area for SC Structures Using Stud and Tie Bar Under Performance Tests

**DOI:** 10.3390/ma17215381

**Published:** 2024-11-04

**Authors:** Yeongun Kim, Byong J. Choi

**Affiliations:** Department of Architectural Engineering, Kyonggi University, Suwon 16227, Republic of Korea; hsyg1444@naver.com

**Keywords:** concrete failure area, pull-out test, SC structure, tie bar, stud, performance test

## Abstract

Nuclear power plants, where steel-plate concrete (SC) structures are commonly adopted, require large-scale components to withstand significant loads, such as those caused by sudden explosions. As a result, SC modular members used in nuclear power plants must have thicker walls filled with concrete compared to standard-sized ones. These large walls also require additional components, such as tie bars and H-shaped steel sections, to reinforce adhesion and resist shear stresses. This study focuses on tie bars placed adjacent to studs and evaluates their influence on the tensile strength of wall structures. To investigate this, we conducted experimental tests using full-scale specimens, including various combinations ranging from single stud to combined stud-tie configurations. Based on the results of these performance tests, we propose a design recommendation for estimating the tensile capacity of SC structures, considering the influence of tie bars.

## 1. Introduction

Steel-plate concrete (SC) structures are currently constructed using steel plates, stud anchors (headed stud anchors), and filled concrete. This construction method is commonly applied to composite bridges that combine both steel and concrete elements as an economical option. To achieve the expected performance of a composite structure, it is necessary to confirm the bonding capability between the concrete and stud materials, which have heterogeneous properties. In SC structures, studs (also known as shear connectors, stud connectors, or headed stud connectors) are welded to steel plates to combine the concrete and surface steel plates [1].

SC structures are formed as sandwich panels that are subjected to bending stresses in many cases. If the bonding strength of each panel is insufficient, separation between the concrete and surface steel plates can easily occur under large shear, tension, moment, or a combination of these forces. Therefore, the concrete breakout strength, shear failure strength of stud connectors, and the maximum strength at the interface between the concrete and surface steel plates are the major forces leading to failure in SC structures. The concrete breakout strength of a single anchor is a failure mode governed by tension, and this strength is also affected by group interference effects in anchors placed close together.

El-Lobody et al. [2] investigated composite girders using a finite element model with 8-node three-dimensional solid elements. Their models predicted deflection behavior and stress distribution along the length of the beam for both solid slabs and precast hollow core elements. Lam et al. [3] examined the shear and load-slip behavior of shear connectors under shear forces. Lin et al. [4] studied the effects of transverse bending moments on stud connections in steel-concrete composite beams, identifying both concrete cone pull-out failure and stud fractures. These failure modes were influenced by stud length under transverse bending moments. Xiang et al. [5] investigated the spacing of studs between concrete slabs and steel girders under transverse bending moments. This study is only tangentially related to the present research, as it focuses on wide shape beams rather than surface steel plates, but it provides insights into the behavior of stud connections under transverse loads.

Badie et al. [6] studied the limits of stud spacing for clusters of studs used in precast concrete panels with wide-shape beams. They performed push-off tests using full-scale composite beams and found that doubling the number of studs per cluster did not significantly affect fatigue. They concluded that full composite action occurred when the spacing between stud clusters was extended to 1220 mm.

J. Qureshi et al. [7] investigated the effects of sheeting thickness and shear connector position on the strength and ductility of shear connectors in long-span composite bridge girders. The authors studied the influence of sheeting thickness and shear stud position on the strength, ductility, and failure modes of the headed shear connector in steel-concrete composite beams. One of the interested variables was the transverse spacing of the shear connectors. Xu et al. [8] also studied the influence of sheeting thickness and shear connector position in composite bridges. Their research focused on the behavior of group studs under biaxial loading in compression. The authors found group studs may lead to comparatively more severe concrete damage at the ultimate stage.

Wang et al. examined the push-out behavior of large stud connectors in steel composite beams. In their study, an empirical shear load-slip equation was proposed, incorporating the maximum stud diameter (25 mm) [9]. Delhomme et al. [10] conducted pullout tests on long anchor bolts, identifying major failure modes such as rod fracture, rod sliding, and cone-shaped concrete breakout. Ballarini et al. [11] studied tension failure mechanisms in rods, including fracture, sliding, and breakout strengths, using 28 specimens to investigate ultimate loads and the relationship between tensile capacity and compressive strength. The variables of the tests were the embedment depth *h*, the support reaction distance *d*, and the age of specimens.

Chen et al. [12] examined anchor bolt failure due to the combined effects of tension and shear. DeVries et al. [13] investigated the anchorage capacity of shallow embedment in concrete. Sartipi et al. [14] studied the tensile response of post-installed anchor connections in both reinforced and plain concrete beams, focusing on key parameters such as embedment depth, section width, and reinforcement effects in post-installed chemical anchors.

Eligehausen et al. [15] researched the behavior and design of adhesive-bonded anchors, identifying potential failure modes such as concrete breakout, mortar-concrete interface failure, and steel-mortar interface failure. Fuchs et al. [16] proposed a behavioral model for concrete breakout failure, which is now incorporated into ACI 318-19 [17]. While concrete breakout failure has been extensively studied, no equations were available to predict the ultimate tensile strength of torque-controlled expansion anchors until Chen et al. [18] proposed a design strength for these anchors.

An experimental investigation on normal concrete to examine the pullout capacity of cast-in-place anchors with embedded circular surrounding studs around rebar under monotonic loading conducted by Turker et al. [19] conducted an experimental investigation into the pullout capacity of cast-in-place anchors with embedded circular studs around rebar under monotonic loading. They carefully studied rebar geometry, steel type, bar spacing, concrete cover, concrete strength, and confinement. Mahrenholtz et al. [20] examined the design standards of post-installed and cast-in-place anchors using both EN 1992-4 and ACI 318. The authors discussed the basic anchor design and underlying production qualification.

Delhomme et al. [21] presented results from pullout tests on large-embedment anchors cast in reinforcement blocks. They studied edge effects and identified failure modes, including concrete cone failure, pullout failure, and combined pullout and concrete cone failure, in both cracked and uncracked sections using headed anchors and ribbed bars.

As described above, most experiments in the literature focus on investigating shear failure of anchors in wide shape beams in bridges. Key factors affecting anchor capacity include anchor length, stud spacing limits in clusters, load application methods, top flange thickness, and group stud effects in wide-shape composite beams. When the large depth in steel plate-concrete structures is needed in critical facilities such as nuclear power plants tie bars that connect top and bottom surface plates are always necessary under the bending, compression, and combination loads. However, few studies have explored the combination of surface steel plates, studs, tie bars, and their combinations.

The paper adopts the concrete cone method in accordance with KCI [22], KDS [23], and ACI 349 [24]. The strength and stress behaviors of adhesive-bonded anchors are discussed, focusing on group and edge effects when studs and tie bars are combined.

The goal of this paper is to identify the maximum tensile loads and group interference effects, with modification factors for steel plate-concrete structures that use both studs and tie bars. As a first step, we focus on the tensile capacity of single and group tie bars in steel plate-concrete structures. We then conduct experiments to evaluate the maximum strength of models using single and group tie bars, simulating the real lateral tensile performance of SC structures using full-scale specimens. This paper primarily focuses on the maximum tensile strength of anchors with the effect of anchor spacing and the combination use of both anchor and tie bars in SC structures. Notably, the maximum tensile strength of the group stud and the combination specimens are experimentally investigated to study the non-overlapped spacing and overlapped spacing effects.

## 2. Experimental Plans

### 2.1. Purposes of Test

Today, in the field of civil engineering and construction, steel-plate concrete (SC) structures are most commonly used in nuclear power plants. These plants are designed to withstand large loads, necessitating the use of large components, such as steel plate-concrete walls filled with thick layers of concrete. To reinforce adhesion and bonding strength in these thick walls, tie bars are used in addition to studs. It is expected that tie bars will enhance the tensile strength of these wall members. However, little research has been conducted on the use of tie bars to estimate the tensile strength of members that combine both studs and tie bars. In light of this, the present report discusses the results of tensile experimental tests to evaluate the influence of tie bars on the tensile strength of SC models. The primary objectives of these performance tests are to review existing design methods for SC structures, particularly focusing on the maximum concrete breakout strength of anchors when both studs and tie bars are used together. Additionally, this paper examines the effective projected concrete failure area of stud groups under the tensile load of single stud specimens. Furthermore, it investigates the projected concrete failure area and proposes a design method that considers the interference effects of tie bars placed near studs.

### 2.2. Design of Specimens

**Figure 1:**
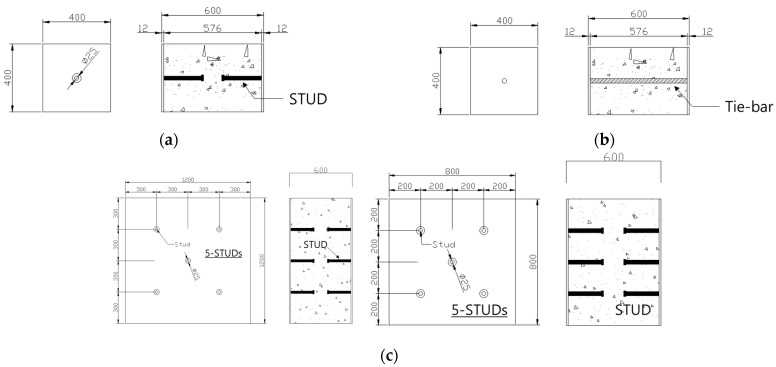

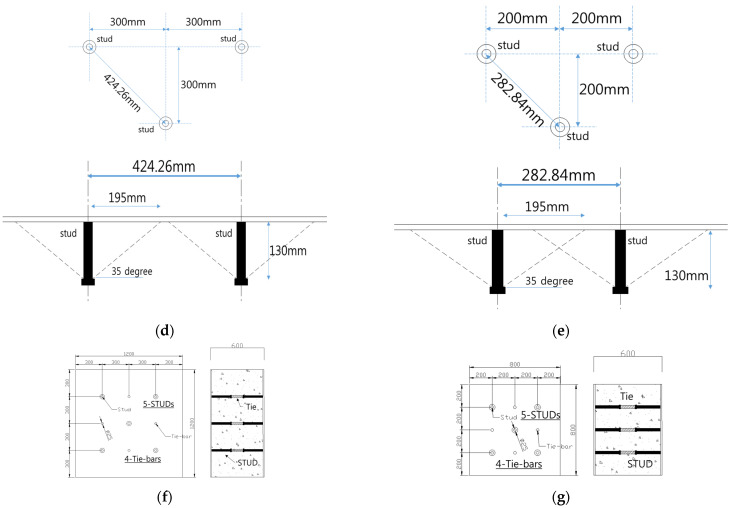
UST, UTI, GST, and GSTS specimen layouts. (**a**) shape of UST (Unit stud) specimen series; (**b**) shape of UTI (Unit tie) specimen series; (**c**) GST (Group Stud) for 200 mm and 300 mm spacings); (**d**) stud layout non-overlapping concrete failure area (GST-1); (**e**) stud layout overlapping concrete failure area (GST-2); (**f**) GSTS-1 with 200 mm spacing; (**g**) GSTS-2 with 300 mm spacing.

To determine the critical tensile load of a stud installed in an SC structure, 12 SC structure specimens were designed for experimental testing. The locations of the studs and tie bars in each specimen were carefully planned to avoid introducing unexpected eccentricities and shear forces during the tests. The specimen categories are as follows: UST (Unit Stud, UST) Series: These specimens were designed to estimate the tensile capacity of an SC member with a single stud. Three specimens were evaluated to obtain average behavior. The dimensions of the UST specimens are 400 mm × 400 mm, as shown in Figure 1a. UTI (Unit Tie Bar, UTI) Series: This series was designed to evaluate the tensile capacity of a tie bar in an SC member. Three specimens were tested to obtain average behavior with the same dimensions as the UST series (400 mm × 400 mm). GST (Group Stud, GST) Series: Two types of GST specimens were designed to estimate the tensile strength of stud groups in SC members. The dimensions of the GST specimens are 1200 mm × 1200 mm, and they include five tie bars. The vertical and horizontal distances between the studs are 300 mm, with a diagonal distance of 424.26 mm, while the second set has a horizontal and vertical distance of 200 mm, with a diagonal distance of 282.94 mm, as shown in Figure 1c,d. GTST (Group Tie and Stud, GTST) Series: This series was designed to assess the influence of tie bars placed among the studs. Two sets of GTST specimens were prepared: GTST-1-A and GTST-1-B: These were designed to examine the effects of non-overlapping configurations with a 300 mm spacing between the studs and tie bars. GTST-2-A and GTST-2-B: These were designed to investigate the effects of overlapping configurations, with a 200 mm spacing between the studs and tie bars. The variables of the specimens used in the experiments are summarized in Table 1. All studs and tie bars in the specimens had a diameter of 25 mm, and the effective length of each stud was 130 mm. For the GST and GTST series, the studs were placed 200 mm and 300 mm apart to test their tensile capacity when the concrete failure areas either overlapped or did not overlap. The layouts of the studs and tie bars for both the GST and GTST series are illustrated in Figure 1c–g.

Figure 1f,g shows plans and sections of the specimens with 5 headed studs and 4 ties. The thicknesses of all specimens are 600 mm. Material tests are conducted before the fabrication of specimens. The test results of material properties on the steel are presented in Table 2, where 3 samples were tested to get the average. The minimum yield strength of 325 MPa was used in all calculations because the minimum yield strength in the tested average showed very little difference that can be disregarded. The compressive stress of concrete used for the specimens is 45 MPa, which is usually used for SC structures. The concrete stress arrived at the strength of 45 MPa after 28 days of curing.

### 2.3. Test Set-Up

**Figure 2:**
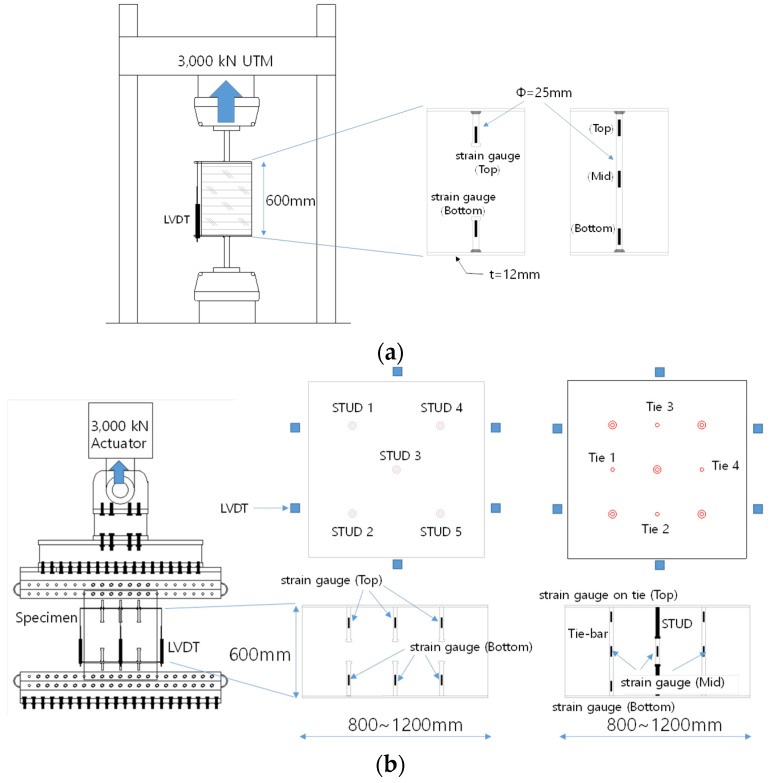
Test set-up of specimens both GST and GTST (**a**) set-up of single stud or tie specimens; (**b**) set-up of stud group specimens.

A set of static tensile loads was applied to the specimens in the vertical direction using a Universal Testing Machine (UTM) with a 10,000 kN capacity. The test setups are shown in Figure 2. The top strong frame was attached to the UTM to evenly support the top of each specimen, while the fixed bottom strong frame was evenly attached to the bottom of the specimen. This setup ensured that the tensile force was evenly transferred to the connection between the testing machine and the specimens. Figure 2 also shows the placement of linear variable differential transformer (LVDT) sensors used to record the displacement of the specimens, as well as the locations where strain gauges were attached inside the specimen.

Reel-type LVDTs were used in the tests and positioned at the same level as the steel plates of the specimens. Strain gauges were directly attached to the studs and tie bars to measure the strains under axial loads. In this study, the UTM was used for the single stud and tie bar specimens (UST and UTI series), while a hydraulic actuator was used for the group stud specimens (GST and GTST series) as the loading equipment. The group stud specimens were set up in a pin-fixed configuration using bolting to prevent internal stresses caused by moments.

## 3. Experimental Test

### 3.1. Results for UST

**Figure 3:**
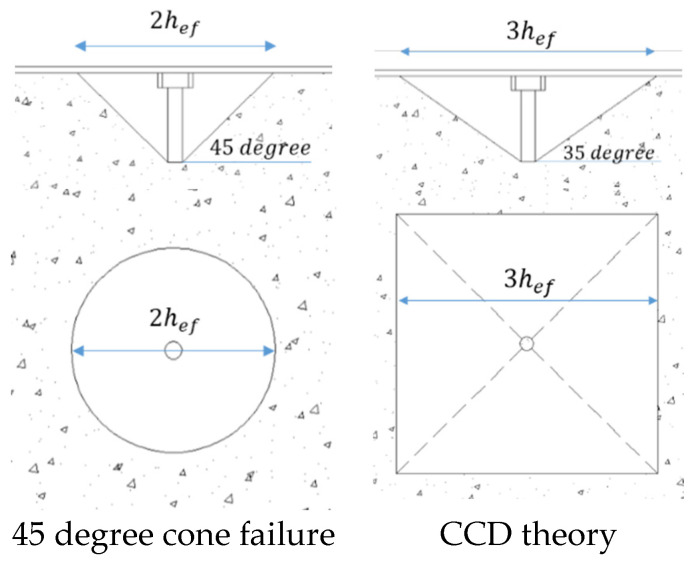
Theories of concrete cone failure.

The studs in SC structures are welded onto the surface steel plates and embedded in concrete. These studs are very similar to headed anchors commonly used in general structures that are anchored to concrete. It is expected that the studs in SC structures will behave similarly to anchors embedded in reinforced concrete (RC) walls. The ACI classifies the failure modes of anchors under tensile loads into five categories: (a) anchor steel failure, (b) anchor pullout, (c) concrete breakout, (d) side-face blowout, and (e) concrete splitting [24]. Concrete breakout failure is significantly influenced by effective embedment depths [24]. For the SC specimen, the concrete around a stud can be considered an infinite medium in the horizontal direction and is located vertically beneath the surface steel plates. Based on these factors, we expect the failure modes of welded-headed studs in SC structures to be limited to three types: (a) anchor steel failure, (b) anchor pullout, and (c) concrete breakout. The expected tensile strength of the stud is calculated using the Concrete Capacity Design (CCD) theory, as specified in KEPIC [25] and ACI 318-19 [17], as illustrated in Figure 3. The nominal tensile strength of an anchor can be expressed using Equation (1) [17]. The design methods for the stud are as follows:

(a) nominal anchor strength:

(1) $$\begin{array}{c} N_{sa}\\ f_{nta} \end{array}\begin{array}{l} =n\cdot A_{se}\times f_{uta}\\ =1\times 490.9\times 446.5=219.2\;kN\\ =min(1.9f_{ya},860\;MPa) \end{array}$$

(b) anchor pullout strength:

(2) $$\begin{array}{cc} N_{p} & =8A_{brg}\times f_{c}^{\prime }\\ & =8\times \frac{\left(37^{2}-25^{2}\right)\pi }{4}\times 45=210.25\;kN \end{array}$$

(c) concrete breakout strength:

(3) $$\begin{array}{cc} N_{b} & =K_{c}\lambda_{c}\sqrt{f_{c}^{\prime }}h_{ef}^{1.5}\\ & =10\sqrt{45}\times 130^{1.5}=99.43\;kN \end{array}$$

Equation (3) is in reference to KDS 14 20 10 [23]. From these results, it is expected that SC structures with a single stud have a concrete breakout failure mode since Equation (3) provides the lowest value.

The results of the load-displacement of the single stud specimens, UST-1, UST-2, and UST-3, are presented in Table 3. According to the results, the average maximum tensile load of all UST specimens is 161.05 kN, and the average tensile load of UST-2 and UST-3 is 163.22 kN. These maximum tensile values are 1.56 to 1.72 times higher than the theoretical code strength of 99.43 kN by Equation (3) (average ratio: 1.62 times).

**Figure 4:**
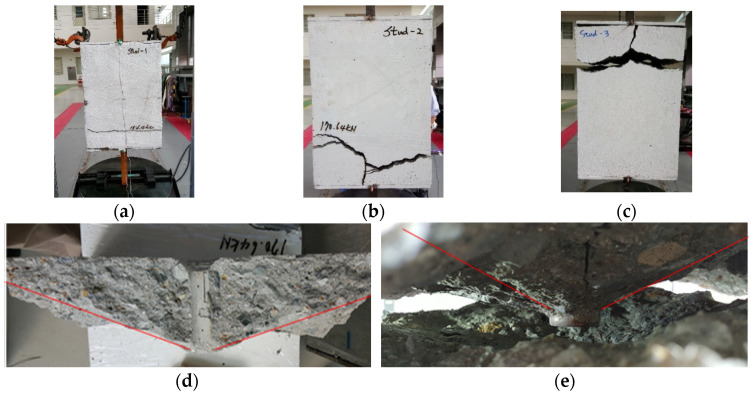
Concrete failure of single stud specimens (**a**) UST-1; (**b**) UST-2; (**c**) UST-3; (**d**) concrete failure of UST-2; (**e**) concrete failure of UST-3.(Red line stands for the crack angle).

Figure 4 illustrates the failure modes of the specimens from the UST model series. From this figure, we can observe that concrete failure occurred on one side rather than symmetrically. It also shows that the failure modes in UST-2 and UST-3 were characterized by cone breakout in cracked concrete, while UST-1 exhibited behavior more indicative of shear failure. In the cases of UST-2 and UST-3, the cone angles were measured at approximately 35 degrees, supporting the assumption that the studs in the SC model behave similarly to anchors as predicted by CCD theory for RC walls.

**Figure 5:**
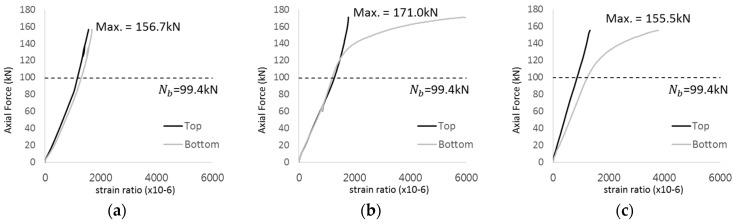
Results of load-strain ratio on stud (UST group) (**a**) UST-1 (**b**) UST-2 (**c**) UST-3.

Figure 5 presents the axial load-strain ratio relationships of a unit stud series from experimental tests. In the UST-1 model, concrete breakout occurred without a significant increase in strain. However, in the UST-2 and UST-3 models, an increase in strain was observed after reaching the tensile strength value $N_{b}$ as shown in Figure 5. The headed studs demonstrated sufficient tensile strength up to the point of breakout failure.

### 3.2. Investigation of UTI Series

The tensile strength of a single-tie bar with a diameter of 25 mm is calculated as follows:

(1) Tensile load by calculation using nominal yield stress = $A_{tie}\times f_{ya}$ = 490.625 × 325 = 159.4 kN
(2) Tensile load by calculation using nominal tensile stress = $A_{tie}\times F_{u}$ = 490.625 × 500 = 245.5 kN
(3) Tensile load by calculation using stress by material tests = $A_{tie}\times F_{u,test}$ = 490.625 × 588.81 = 289.03 kN

**Figure 6:**
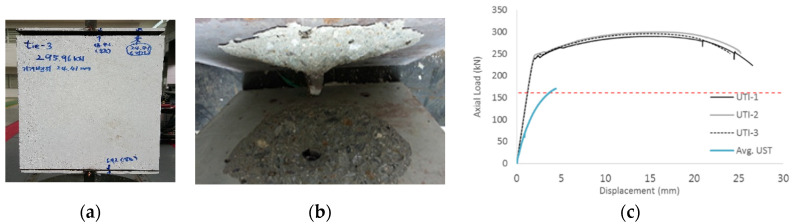
Failure shape and load vs. vertical displacement on UTI group. ((**a**): exterior shape, (**b**): tie and concrete fractures, and (**c**): axial load-displacement relationship).

The failure shape of the UTI specimens is shown in Figure 6a,b, where cone failure occurred near the tie bar. Both the concrete and the tie bar failed simultaneously without a distinct indication. The results of the single tie bar specimens (UTI-1, UTI-2, and UTI-3) are presented in Figure 6 and Table 4. These results show that the tensile strengths of UTI-1, UTI-2, and UTI-3 are close to the values calculated using Equation (3) with the actual tested material strength. The horizontal dotted lines in Figure 6c represent the average maximum tensile load of 161.05 kN from the UST (Unit Stud Series) specimen group. The maximum tensile loads of the UTI specimens exceed 161.05 kN, indicating that a single tie bar has greater tensile strength than a single stud of the same diameter. It was observed that the tie bar, directly welded to the surface steel plates, exhibited full plastic behavior after the concrete cracked. No slippage due to friction loss between the concrete and tie bars was observed, as shown in Figure 6. In Figure 6a,b, the cone shape of failure mode around the tie bar appeared at the final stage of failure.

**Figure 7:**
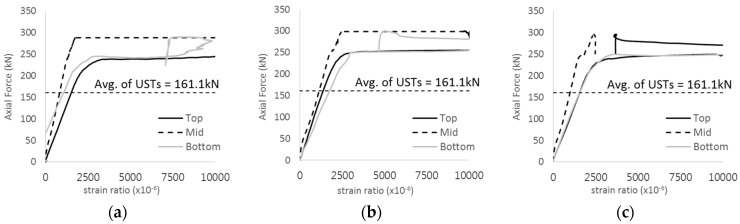
Results of load-strain ratio on tie bar (UTI group) (**a**) UTI-1, (**b**) UTI-2, and (**c**) UTI-3.

The axial force versus strain ratio relationships for the tie bars are shown in Figure 7. Although some abnormal values were recorded due to concrete cracking, almost all tie bars exhibited stable plastic behavior with consistent elasto-plastic stiffness.

### 3.3. Investigation of a Stud Group (GST Specimens)

The purpose of the GST specimens, featuring a stud group, is to evaluate both the tensile strength of the stud group and the effects of overlapping concrete failure areas between the studs. The stud spacing distances in the GST series are 300 mm for GST-1 and 200 mm for GST-2, respectively, shown in Table 5. The GST-2 specimen, with a stud spacing of 200 mm, specifically aims to investigate the overlapping effect of concrete breakout at the ultimate strength stage.

The concrete failure area of a stud group is determined as shown in the bottom of Table 5. These areas are calculated by the CCD method using 35 degree triangular pyramids. The hatched areas indicate the effective areas. $A_{nc}$ is the total projected concrete failure area of a single anchor or group of anchors, while $A_{Nco}$ is the projected concrete failure area of a single anchor with an edge distance equal to or greater than ${1.5h}_{ef}$ [17]. In this case, $A_{Nco}={9h}_{ef}^{2}=152,100$ mm^2^.

(4) $$N_{cbg}=\frac{A_{Nc}}{A_{Nc0}}\psi_{ec,N}\psi_{ed,N}\psi_{c,N}\psi_{cp,N}N_{b}\;$$

(5) $$A_{Nc0}=\left(2\times 1.5h_{ef}\right)\times \left(2\times 1.5h_{ef}\right)\;$$

**Table 5:** Comparison of effective concrete failure area of GST-1 and GST-2 or the GST-1 specimen, where the concrete failure areas of the studs do not overlap, the ratio ($n_{eff}$) of the concrete failure area is 5 times larger than that of a single stud. In contrast, for the GST-2 specimen, the failure area ratio is 3.6 times larger than that of a single stud due to the overlapping effects. In the case of GST-2, the effect of multiple anchors on the nominal concrete breakout strength in tension reached about 72% of the concrete failure area ratio of GST-1. The nominal concrete breakout strength under tensile loads, calculated using Equation (4) (ACI and KEPIC), is shown in Table 6. Here, $N_{b}$ is 99.43 kN by Equation (3) and 161.05 kN by the average tensile load of the UST series specimens with a single stud.

GST-1 at 300 mm Spacing, Col (a)	GST-2 at 200 mm Spacing, Col (b)
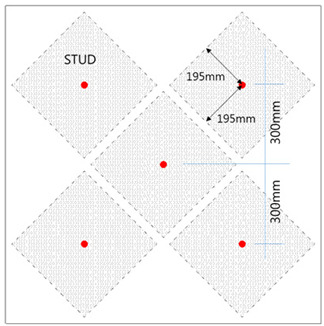	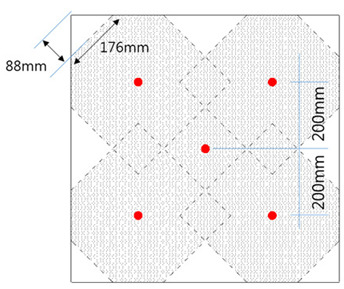
$\frac{A_{Nc}}{A_{Nco}}=5=n_{eff}$	$\frac{A_{Nc}}{A_{Nco}}=3.6=n_{eff}$
$A_{Nc}=760,500\;{mm}^{2}$	$A_{Nc}=547,000\;{mm}^{2}$

**Figure 8:**
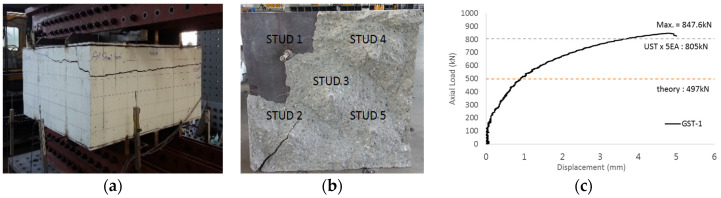
Tests of specimen GST-1. ((**a**): side view, (**b**):inside, and (**c**):load-displacement relationship).

The overall shape and failure pattern of GST-1 are shown in Figure 8a,b. Figure 8c presents the load-displacement relationship of GST-1 from the performance test. The ultimate failure, as seen from the side view, resembles a wave, with the interior exhibiting cone-shaped failures in studs 2, 3, 4, and 5. The displacement values in Figure 8 represent the average of the six LVDTs, which recorded the vertical displacement of GST-1 with 300 mm stud spacing. From the figure, the ultimate load of the GST-1 specimen at failure reached 847.6 kN, approximately 1.7 times greater than the calculated nominal concrete breakout strength in tension, which is 497 kN. The maximum ultimate load at failure was also 1.05 times higher than the 805 kN recorded for the UST specimen, based on the average tensile breakout strength.

**Figure 9:**
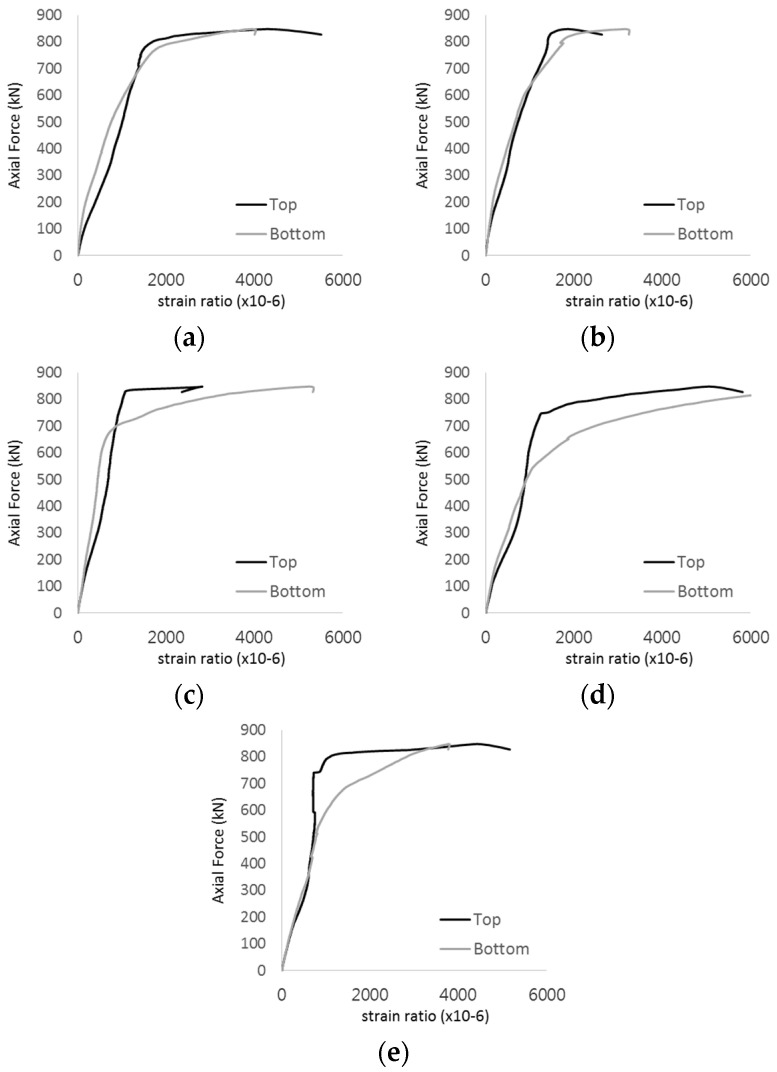
Histories of strain ratios of GST-1 (**a**) STUD 1, (**b**) STUD 2, (**c**) STUD 3, (**d**) STUD 4, and (**e**) STUD 5.

The strain ratios of the studs in GST-1 under axial tensile loading are shown in Figure 9. From the figure, it is observed that the studs exhibit plastic behavior starting at around 800 kN. At the load level of 497 kN, corresponding to the theoretical concrete breakout strength, all studs remained in the elastic strain state. In Figure 9c, the bottom line shows a slight upward shift, likely due to an initial adjustment of the LVDT with the testing machine. The studs located at the bottom of the specimen initially displayed plastic behavior, similar to the results of the UST series specimens with a single stud. These plastic states developed in a bi-linear fashion until the specimen reached failure. It is also noted that the studs generally exhibited consistent behavior, indicating that the tensile load was evenly distributed among all studs during the experimental test.

**Figure 10:**
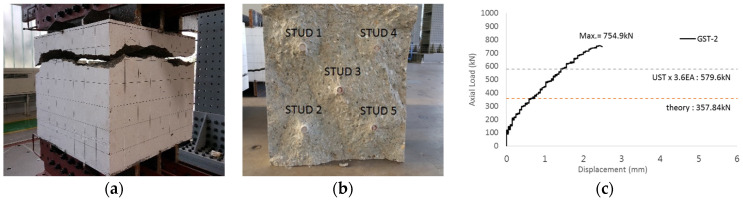
Result of failure pattern (GST-2). ((**a**): top failure, (**b**): inside, and (**c**): load-displacement relationship).

The GST-2 specimen was designed to investigate the overlapping effect during concrete failure stages. The concrete failure area of the GST-2 specimen was calculated to be approximately 72% of the area of GST-1, due to the reduced spacing between studs. This also corresponds to a modification factor of 3.6 for the studs. The overall failure pattern, characterized by cone-shaped breakouts around the studs, is shown in Figure 10a,b where the cone failure modes appeared in all five studs. The experimental performance test result for GST-2 is illustrated in Figure 10c. Similar to GST-1, the axial load versus displacement relationship of the GST-2 specimen, shown in Figure 10, was determined from the average displacement values recorded by six LVDTs. The ultimate load of GST-2 at failure was 754.9 kN, about 89% of the ultimate load of the GST-1 specimen, where the concrete failure areas of the studs did not overlap. This load is also about 2.1 times greater than the nominal concrete breakout strength of 357.84 kN, as calculated using Equation (4), and 1.3 times greater than the average breakout strength in tension for the UST series (579.6 kN). Notably, no plastic flow was observed in the GST-2 specimen.

**Figure 11:**
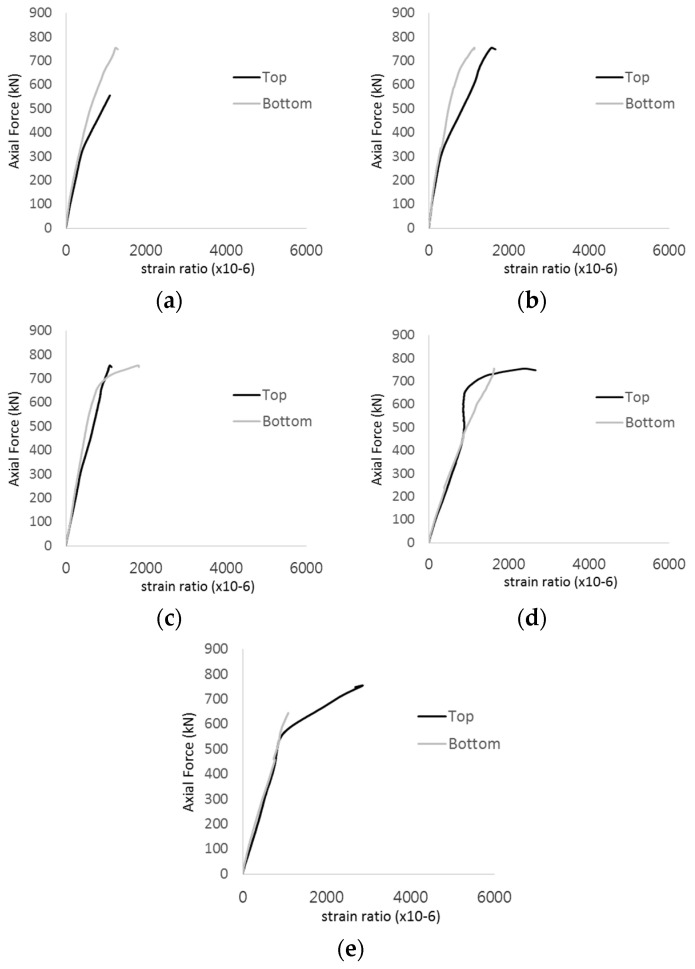
Histories of strain ratios of GST-2 (**a**) STUD 1, (**b**) STUD 2, (**c**) STUD 3, (**d**) STUD 4, and (**e**) STUD 5.

The historic strain ratios of the studs in the GST-2 specimen are shown in Figure 11. The results indicate that concrete failure occurred while the studs remained in their elastic states. This observation aligns with the results from the UST and GST-1 specimens. In contrast, the specimen with non-overlapping concrete failure areas (GST-1) exhibited full plastic deformations, highlighting the impact of stud spacing on the load distribution and failure behavior.

The tensile loads of the GST specimens from the performance tests were approximately 1.3 times higher than the theoretical breakout strength in tension, which is consistent with the behavior observed in SC specimens with a single stud from the UST series. Based on the comparison of performance test results, the CCD theory proves to be an effective method for estimating the tensile capacities of SC structures containing only studs. Studs with non-overlapping concrete failure areas exhibit some plastic behavior and higher tensile capacities. However, in cases where the concrete failure areas overlap, cracking in the concrete occurs before the studs reach their maximum tensile capacities due to the interference effects between adjacent studs.

### 3.4. Investigation of GTST Specimens with Studs and Tie Bars

**Figure 12:**
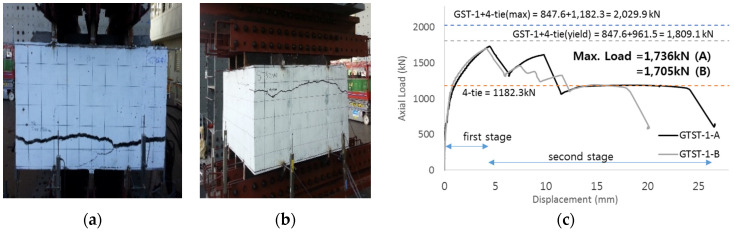
Result of GTST-1 (**a**) bottom (**b**) top (**c**) load-displacement relationship.

SC structures require tie bars for reinforcement. These tie bars are welded to steel surface plates that cover the SC structures and are placed adjacent to the studs. Previous research has indicated that when studs and tie bars are placed together, their tensile capacities are mutually influenced [19]. Based on these findings, this paper conducts experimental performance tests on specimens with both studs and tie bars to confirm the interaction between them. It also proposes a design method for estimating the tensile loads of SC structures, taking this interaction into account. The GTST-1 series specimens, containing both tie bars and studs, were designed to ensure that the concrete failure areas of the studs do not overlap. The maximum tensile loads for the GTST-1 series can be derived using the following cases: Case (a) is the strength of both the tensile load of studs and tie bars based on nominal yield stress: 99.4 × 5 + 159.4 × 4 = 1134.6 kN. Case (b) is the strength of both the average maximum tensile load of USTs and the yield load of UTI tests: 161 × 5 + 240.37 × 4 = 1766.48 kN. Case (c) is the maximum tensile load of GST-1 with yield load of UTIs: 847.6 + 240.37 × 4 = 1809.08 kN. Case (d) is the maximum tensile load of GST-1 with tested strength of UTIs: 847.6 + 295.57 × 4 = 2029.88 kN The overall failure patterns at the top and bottom are illustrated in Figure 12a,b. The picture does not show the inner face of concrete because the specimens are too thick to break after the experiments. Figure 12c shows the load-displacement relationship for the GTST-1 series specimens. The maximum tensile loads recorded from the experimental results were 1736 kN for GTST-1-A and 1705.1 kN for GTST-1-B. The average maximum strength for the GTST-1 series, where non-overlapping spacing was maintained, was 1720.5 kN.

Table 7 presents the difference ratios between the strengths from the test results and the expected calculations, based on the four assumptions (a) through (d). The ratios are calculated by dividing the test results by the predicted loads. From Table 7, it is observed that, except for case (a), all calculated loads exceed the actual tensile strengths obtained in the performance tests. This indicates that the assumptions in cases (b), (c), and (d) tend to overestimate the real tensile strength of the specimens.

The first load degradation in the GTST-1 specimens occurred at a displacement of approximately 4 mm, as shown in Figure 12. This behavior closely aligns with the GST-1 specimens, which contain only studs, showing an ultimate displacement of 4 to 5 mm before concrete failure. The load degradations in GTST-1 appear in steps, unlike GST-1, where the load decreases more uniformly. It is assumed that the gradual reduction in stud strength in GTST-1 is due to the proximity of the tie bars, whereas the strength of the studs in GST-1 decreases simultaneously. Finally, in the GTST-1 series, the plastic plateau reaches the same total load as the sum of the tensile loads of the tie bars following the concrete failure.

Table 8 presents the strain ratio histories of the studs 1, 2, 3, 4, and 5 in the GTST-1-A and GTST-1-B specimens. The strain histories are divided into two stages: the first stage records strains up to the initial failure load, and the second stage records strains leading to the final breakage of the specimens. In other words, the first stage is to investigate the load level and strain up to yield regions of all studs. The second stage shows all strain behaviors up to the final stages. When comparing the failure load of GST-1 (group stud only without tie bars) to the first failure load of GTST-1, the strain histories of the studs in GTST-1 resemble more closely those in GST-2 (group stud-2 specimen) than the ones in GST-1 (group study-1 specimen), despite having the same stud arrangement. All strains of stud showed the plastic flow except stud 3 in the GTST-2 series because of the uneven load distribution according to the failure of concrete shown in the second of Table 8 for stud number 3. From Table 8, one could find the general plastic strains of studs. It is also observed that an unloading phenomenon occurs in the studs during the second stage, indicating that after the concrete cone failure, the studs lose tensile resistance to cease further tensile deformation. This confirms that after concrete failure, only the tie bars continue to bear the tensile loads in the SC specimens.

Table 9 presents the strain ratio histories of the tie bars in the GTST-1-A and GTST-1-B specimens. The tie bars exhibited bi-linear plastic behavior in both the top and bottom ties prior to the concrete cone failure shown in tie bars -1,2,3, and 4 through the second stages. The pullout load is not fully distributed on the tie bar 2 of the GTST-1-A specimen in Table 9, tie bar 2. Based on these findings from the GTST-1 specimens, the median value between the yield and maximum tensile load is conservatively considered the tensile strength of a tie bar. The tensile load is evenly distributed to the top and bottom tie bars from Table 9 tie bars 1, 3, and 4.

### 3.5. Results of GTST Specimens with Tie Bars and Studs Having Overlapping Concrete Failure Areas

The GTST-2 A and GTST-2 B specimens, with tie bars and studs overlapping in spacing, were evaluated in comparison to the GTST-1 series. To estimate the maximum tensile strength for the GTST-2 specimens, the following assumptions were made: (a) tensile load of studs and tie bars using nominal tensile stress = 99.4 × 3.6 + 159.4 × 4 = 995.44 kN; (b) average maximum tensile load of USTs + yield load of UTIs = 161 × 3.6 + 240.37 × 4 = 1541.08 kN; (c) maximum tensile load of GST-2 + yield load of UTIs = 754.9 + 240.37 × 4 = 1716.38 kN; and (d) maximum tensile load of GST-2 and UTIs = 754.9 + 295.57 × 4 = 1937.18 kN.

**Figure 13:**
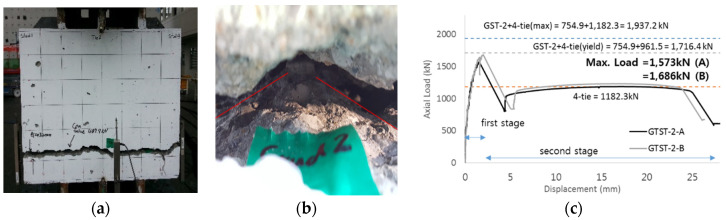
Result of GTST-2. ((**a**): bottom failure, (**b**): inside, and (**c**): load-displacement relationship).

The overall failure pattern of the GTST-2 specimens is shown in Figure 13a,b, where a cone failure pattern was observed. The axial load vs. displacement relationships for the GTST-2 series are presented in Figure 13c. The maximum tensile loads for GTST-2-A and GTST-2-B were recorded as 1573.3 kN and 1685.9 kN, respectively, yielding an average maximum tensile strength of 1629.5 kN for GTST-2 (with overlapped spacing). This represents 94.7% of the tensile strength of GTST-1 (1720.5 kN), indicating that the non-overlapped specimens of GTST-1 exhibited 5.3% higher tensile strength compared to the overlapped specimens of GTST-2.

Similar to Table 7, Table 10 highlights the differences between the calculated and tested tensile strengths. For GTST-2, with the exception of assumptions (a) and (b), the calculated values exceeded the experimental results, indicating that the calculations overestimated the actual tensile strength for the specimens with overlapping spacing.

The maximum tensile load of GTST-2-B is slightly higher than that of GTST-2-A. The load degradations for GTST-2-A and GTST-2-B occurred at displacements of 1.565 mm and 2.003 mm, respectively, which are lower than the maximum displacement of 2.49 mm observed in the GST-2 series. This suggests that the additional interference effect present in the GTST-2 specimens did not occur in the GST-2 (overlapping) specimen. In GTST-2, after the first concrete failure, the tie bars bear the tensile load, resulting in platform tensile loads that are almost equivalent to the sum of the tensile strength of the tie bars, similar to the GTST-1 specimens. However, no stepped load degradations were observed in the GTST-2 specimens. These findings indicate that the tie bars, located closer to the studs in the GTST-2 specimens, had a greater impact on the concrete failure areas compared to those in the GTST-1 specimens.

Table 11 presents the strain ratio histories of the studs in GTST-2-A and GTST-2-B. From the table, it is evident that the studs in the GTST-2 specimens remained in an elastic state until the occurrence of concrete cone failure. This indicates that, in the case of the GTST-2 specimens, the concrete cone failures happened prior to the failure of the studs, resulting in maximum tensile strengths that are lower than those of the GTST-1 specimens.

Table 12 presents the strain ratio histories of the tie bars in the GTST-2-A and GTST-2-B specimens. Similar to the GTST-1 specimens, the tie bars in the GTST-2 specimens exhibit bi-linear plastic behavior. It is observed that the edges of the tie bars were already in a plastic state at the point of the first load degradation.

## 4. Suggestion for Estimating the Tensile Ability of SC Structures Having Studs and Tie Bars

Based on the performance tests of the GTST specimens, the tensile failure of SC structures with studs and tie bars can be divided into two stages. The first stage involves concrete cone failure around the studs due to the loss of concrete friction, while the second stage involves the yielding of the tie bars welded to the cover steel plates. The first stage occurs when the concrete around the studs deforms beyond its elastic limit due to the elongation of the studs, leading to cracking. However, in specimens with closely spaced studs, such as GST-2, concrete cone failure was observed earlier, likely influenced by adjacent studs, before the studs themselves lost elasticity. Similar phenomena were observed in SC specimens where the studs were spaced sufficiently apart, but the tie bars were positioned within the concrete failure zone of the studs. Therefore, it can be assumed that the tensile strength of SC structures is influenced by both the tie bars and the studs.

**Figure 14:**
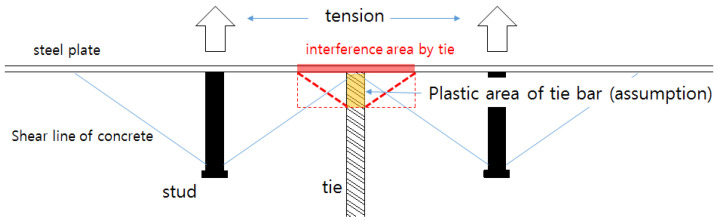
Interference area caused by stud and tie under tension (SC structure).

According to Table 9 and Table 12, it is observed that only the edges of the tie bar yield from top view, while the elasticity at the middle of the tie bar remains under tension. From these results, this paper presents the failure mechanism as shown in Figure 14.

Figure 14 illustrates that the elastic behavior of the concrete around the studs is lost under maximum tensile loads, leading to the formation of shear lines [16]. Simultaneously, the tie bars placed between the studs elongate beyond their yield limit, causing concrete cone failure within the interference area of the tie bars—similar to what occurs around the studs. Considering this, the concrete failure area of SC structures with studs and tie bars can be calculated using the same method as SC structures with only studs. It is thus assumed that the maximum tensile load is lower than the expected load if the concrete around the tie bars retains its elasticity. The proposed method for estimating the tensile capacity of SC structures with both studs and tie bars is based on the ACI 318 [17] and KEA [25] guidelines for determining anchorage concrete breakout strength under tension. This report defines the effective plastic length of tie bars under tension as the length of the tie bar in a plastic state. In previous research on anchors in general structures, shallow embedment is typically defined as 3–5 times the bar diameter. The main categories of this model are concrete cone failure and the concrete capacity model. The embedment length (effective plastic length) is directly related to anchor failure. In this paper, the effective plastic length of tie bars is calculated by applying the following assumptions:

(a) The elastic strain ratio of each tie bar is equal to the strain ratio in the middle of the tie bar.
(b) The second stiffness (plastic stiffness) of the tie bars at the edges is equal.

**Figure 15:**
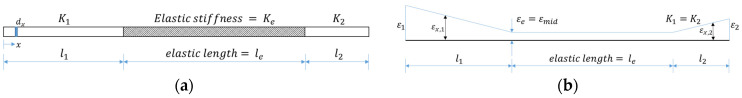
Concept of stiffness and strain in tie bar under tension (total length is 600 mm, D = 25 mm). ((**a**): concept of effective length and (**b**): strains with elastic length).

The mechanism and method for calculating the effective plastic length, $\delta_{i},$ are presented in Figure 15 and Equation (6).

(6) $$\delta_{i}=\varepsilon_{e,i}\times l_{i}+\int_{0}^{l_{1}}\left(\varepsilon_{x,1}-\varepsilon_{mid}\right)dx+\int_{0}^{l_{2}}\left(\varepsilon_{x,2}-\varepsilon_{mid}\right)dx=\varepsilon_{e,i}\times l_{i}+\int_{0}^{l_{1}+l_{2}}\left(\varepsilon_{x,1}+\varepsilon_{x,2}-2\varepsilon_{mid}\right)dx\;=\varepsilon_{e,i}\times l_{i}+\frac{1}{2}\left(\varepsilon_{1,i}+\varepsilon_{2,i}-2\varepsilon_{mid,i}\right)\left(l_{1}+l_{2}\right)$$

The effective plastic length, $\delta_{i}$, over the variable length *x* x, can be summarized through algebraic integration by hand. In Equation (6), it is assumed that the ratio of length is proportional to the ratio of strain, which leads to Equation (7). The strain ratios used in Equation (6) are derived from experimental results. The theoretical lengths, *l* 1 l 1 and *l* 2 l 2, vary and change based on the different strain values observed in the tests.

(7) $$l_{1}:l_{2}=\varepsilon_{1}:\varepsilon_{2},\frac{l_{1}}{l_{2}}=\frac{\varepsilon_{1}}{\varepsilon_{2}}$$

The effective plastic lengths of the tie bars, calculated using Equations (6) and (7), are presented in Table 13 and Table 14. Table 14 provides the effective plastic lengths based on the diameter of the tie bar. In these cases, it is assumed that the tension applied to the tie bar is proportional to the strain ratio of each tie bar. Additionally, the average displacement of the tie bars is considered equal to the displacement of the specimen in which the tie bars are embedded.

From Table 14, the effective plastic lengths of the tie bars in GTST-1s, in which the studs are placed so that the concrete failure areas between the studs do not overlap, are 4.56 times the diameter of the tie bars on average. In GTST-2s, in which the studs are placed so that the concrete failure area overlaps that of other studs, the effective plastic lengths are about 1.94 times the diameter of the tie bars on average. Therefore, it is necessary to think of the ranges between 2D through 5D (conservatively) for practical design purposes.

**Figure 16:**
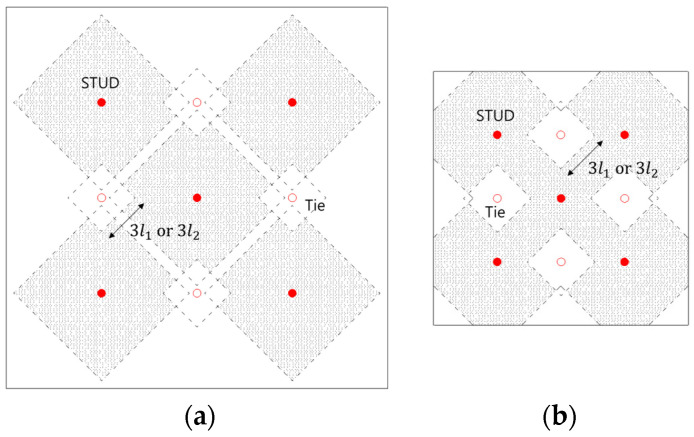
Effective concrete failure area of studs considering tie bars (**a**) GTST-1s (**b**) GTST-2s, (red solid is for the center of group).

Figure 16 presents the suggested effective concrete failure areas, indicated with hatching, for the GTST-1 and GTST-2 specimens, accounting for the influence of tie bars. These effective areas exclude the regions affected by the tie bars’ interference. The proposed concrete failure areas for the stud group, along with the effective number of studs based on the effective plastic length of tie bars at 2D, 3D, and 5D, are outlined in Table 15. For calculating the tensile strength of the anchors while considering the effect of tie bars, the projected concrete failure area is denoted as $A_{Nc.t}$ as shown in Equation (8), where the 9($l_{1or2}^{2}$) was used instead of 9$h_{ef}^{2}$, and the number of effective studs considering the tie bars’ effect is defined as $n_{eff,s}$ as shown in Equation (9).

(8) $$A_{Nc.t}=A_{Nc}-n_{tie}\times 9l_{1or2}^{2}$$

(9) $$n_{eff,s}=\frac{A_{Nc,t}}{n_{s}\times A_{Nc0}}$$

Equation (10) presents the suggested method for calculating the tensile load of SC structures with studs and tie bars, while Equation (11) compares this method to the results from performance tests. The performance tests revealed that numerous tie bars were in a plastic state at the maximum tensile load. However, for safety in design, Equations (10) and (11) conservatively apply the average yield load of UTIs (240 kN). The maximum tensile load of a single stud in SC structures $N_{b,test}$, used in Equation (10), corresponds to the average yield load of USTs (161 kN). The area $N_{b,test}$, in Equation (11) is the area calculated by accounting for the influence of tie bars, as shown in Table 16.

(10) $$N_{b}=N_{b,test}\times n_{eff,s}+N_{y,tie}\times n_{tie}$$

(11) $$N_{b}=\mathrm{maximum}\;\mathrm{tensile}\;\mathrm{load}\;\mathrm{of}\;\mathrm{GSTs}\times \frac{A_{Nc,t}}{A_{Nc}}+\mathrm{average}\;\mathrm{of}\;\mathrm{yield}\;\mathrm{load}\;\mathrm{of}\;\mathrm{UTIs}$$

Table 16 provides a comparison between the suggested method and the test results by dividing the test values by those derived from the assumptions, similar to Table 10. In Table 15, if one can get modified $N_{b}$ using Equations (10) and (11), the one can evaluate various failure cases once more by the normalization using 1.0. From the comparison, the case using an effective plastic length of 2D for the tie bars most closely aligns with the performance test results. Meanwhile, using a plastic length of 3D proves to be the most effective for safety considerations in design. The three calculations (2D, 3D, and 5D) based on test data simplify the maximum load-bearing strength for the GTST specimens, allowing for comparison under assumptions such as (a) tensile load of studs and tie bars using nominal tensile stress, (b) average maximum tensile load of UST and yield load of UTI, and (c) maximum tensile load of GST-2 and yield load of UTI, as seen in Table 10, where effective length is represented by diameter instead of $h_{ef}$.

The results indicate that both Equations (10) and (11), along with the assumptions, are reasonable. The comparison ratios for the 2D effective length show a range from 0.99 to 1.17, making it a good estimate for practical application. Based on the comparison of the ratio between test results and Equations (10) and (11), the suggested method using effective plastic length can cautiously be applied to non-overlapping anchors, with ratios ranging from 1.01 to 1.03. For overlapping anchors, however, the ratio is higher, between 1.29 and 1.46, indicating that the test values are too high to adopt the maximum strength ratio using 2D and 3D effective lengths.

From these findings, this paper suggests that if the tensile capacity of a stud in SC structures is assured, it is possible to estimate the tensile load of SC structures accurately using the proposed method in Equation (10).

## 5. Conclusions

Based on the performance tests using SC structure specimens with studs and tie bars, the following conclusions could be drawn:

(1) In SC structures, studs that are spaced apart so that the concrete failure areas between them do not overlap exhibit maximum tensile capacity up to the point of concrete cone failure, as predicted by the CCD theory. However, in specimens where the concrete failure areas of the studs overlap, cracks form before the studs reach their maximum tensile strength due to the concrete cone failure from neighboring studs.
(2) The non-overlapped GTST-1 specimens, with both studs and tie bars, demonstrated 5.3% higher tensile strength compared to the overlapped GTST-2 specimens. This reduction in strength in GTST-2 is attributed to the tie bars creating overlapping failure areas between the anchors. Even when the studs are spaced sufficiently apart to avoid overlapping concrete failure areas, cone failure can still occur before the studs reach their maximum strength if tie bars are positioned within the failure zone of the studs.
(3) To account for this interference, the method for estimating the tensile strength of SC structures excludes the areas affected by the tie bars. This paper recommends the following effective plastic lengths for tie bars to avoid interference: 2 times the diameter of the tie bar (2D) for calculating the actual tensile loads of SC structures. 3 times the diameter of the tie bar (3D) for designing with safety considerations, particularly for non-overlapping anchors.

The proposed method has been validated using performance tests on SC structures with individual studs. Consequently, if the performance of a single stud in an SC structure is standardized, this method can be effectively applied in general design for non-overlapping anchors. Further studies are necessary to refine the assumptions for overlapping anchor systems.

## Figures and Tables

**Table 1:** Experimental Schedule with stud and tie bar.

Specimen Types	Number		Space (mm)	Depth t (mm)	Overlap, Non-Overlap
	Stud	Tie Bar			
UST-1 (Unit Stud)	1	-	-		N.P
UST-2 (Unit Stud)	1	-	-		N.P
UST-3 (Unit Stud)	1	-	-		N.P
UTI-1 (Unit Tie bar)	-	1	-		-
UTI-2 (Unit Tie bar)	-	1	-		-
UTI-3 (Unit Tie bar)	-	1	-		-
GST-1 (Group Stud)	5	-	300	600	N.P
GST-2 (Group Stud)	5	-	200		O.P
GTST-1-A (Group Tie and Stud)	5	4	300		N.P
GTST-1-B (Group Tie and Stud)	5	4	300		N.P
GTST-2-A (Group Tie and Stud)	5	4	200		O.P
GTST-2-B (Group Tie and Stud)	5	4	200		O.P

**Table 2:** Tensile stress of steel materials (MPa, mm).

Member	D, mm	t, mm	$F_{ya}$	$F_{u}$	$F_{y,test.avg}$ of 3 Coupons	$F_{u,test.avg}$ of 3 Coupons
Headed Stud	25	-		400	325.04	401.53
Tie bar	25	-	325	500	325.09	588.81
Steel plate		12 mm		500	325.04	543.87

**Table 3:** Maximum tensile load of UST models.

	Max. Load (kN)	(Max. Load)/(Equation (3) by ACI 318) [12]
UST-1	156.7	1.58
UST-2	171.0	1.72
UST-3	155.5	1.56
Avg.	161.05 (≈161.1)	1.62

**Table 4:** Maximum tensile load of UTI group.

	Tensile Load (kN)	
	At Yield	At Max.
UTI-1	232.29	290.29
UTI-2	248.90	299.93
UTI-3	239.93	296.49
Avg.	240.37	295.57

**Table 6:** Effective tensile breakout strength of group studs.

$N_{cbg}$		GST-1 (5 ea)	GST-2 (3.6 ea)
Cal.	$N_{b}:$ by Equation (3). ($N_{b}={k_{c}\lambda }_{c}\sqrt{f_{c}^{\prime }}h_{ef}^{1,5}$)	497.0 kN	357.8 kN
	$N_{b}:$ use avg. of UST (161.05 kN)	805.0 kN ${(n}_{eff}=5$)	579.6 kN ${(n}_{eff}=3.6$)
Strength by experimental test		847.6 kN	755.0 kN

**Table 7:** Results comparison (test/assumption).

Assumption	GTST-1-A	GTST-1-B	Avg.
(a)	1.53	1.50	1.52
(b)	0.98	0.97	0.98
(c)	0.96	0.94	0.95
(d)	0.86	0.84	0.85

**Table 8:** Histories of strain stages for studs (strain ratio, GTST-1).

**STUD**	**GTST-1-A**	
	**First Stage**	**Second Stage**
1	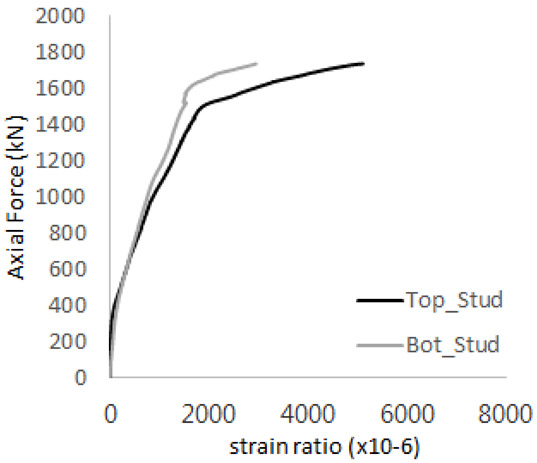	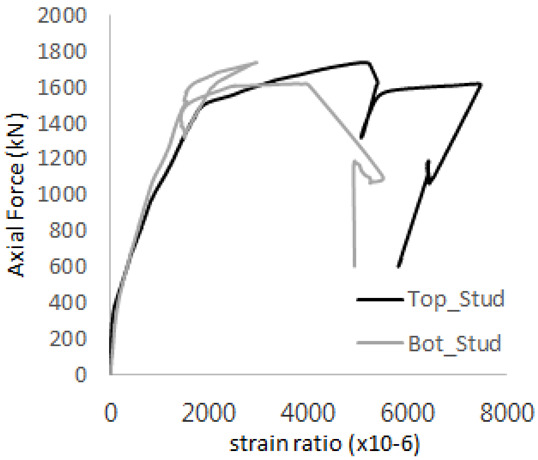
2	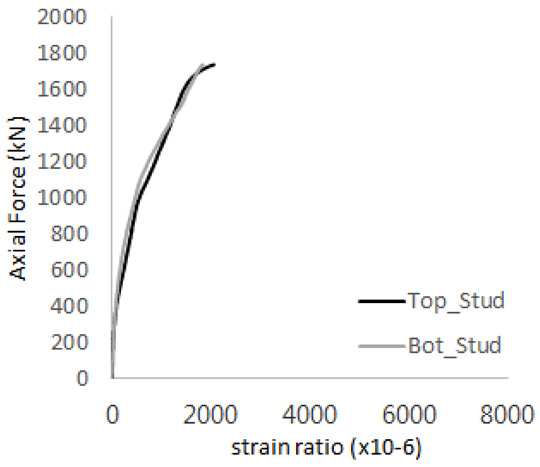	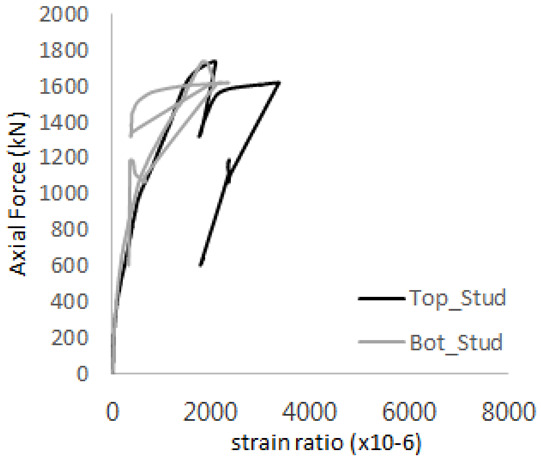
3	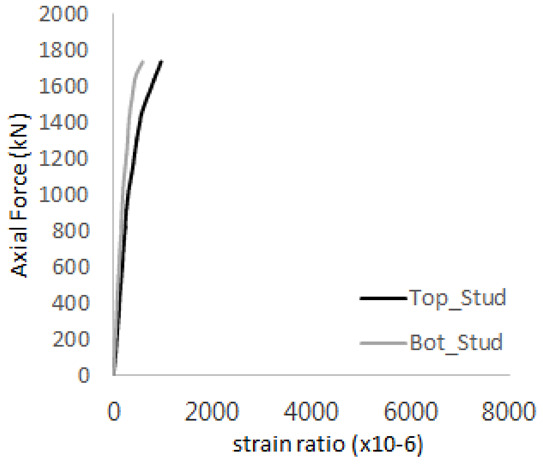	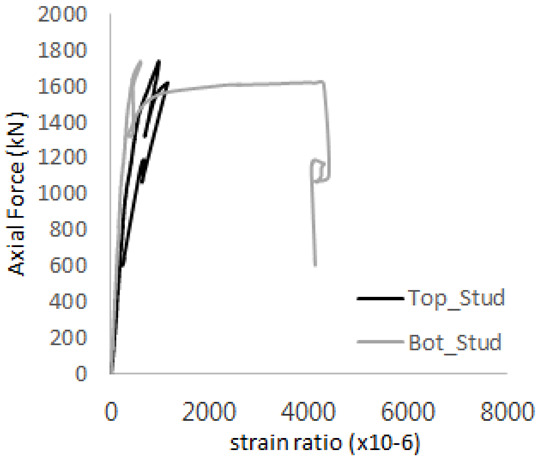
4	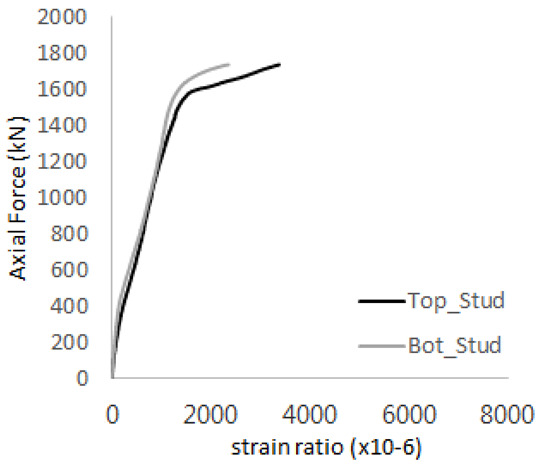	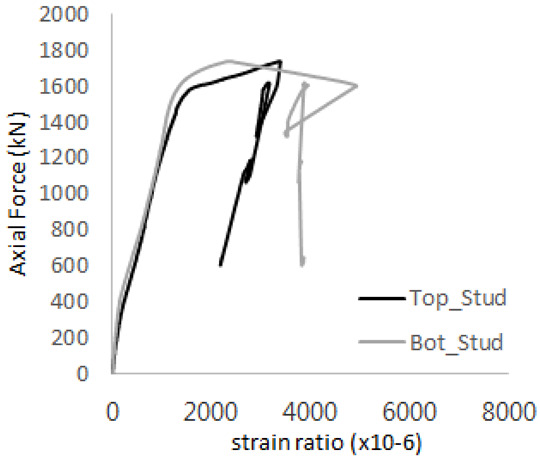
5	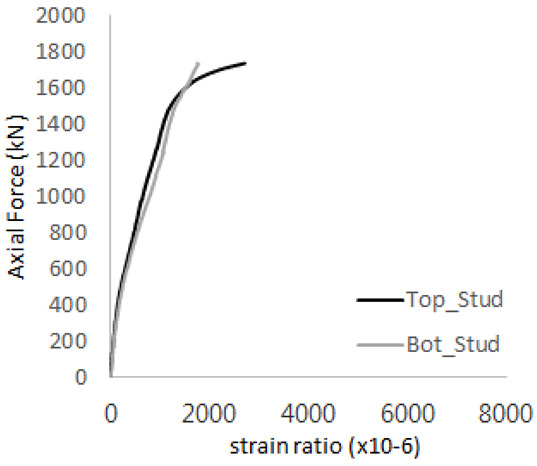	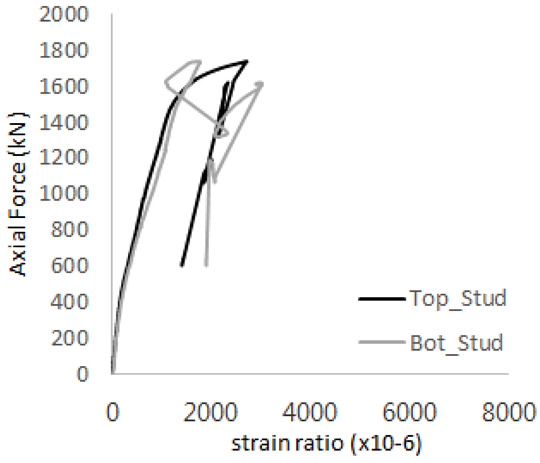
**STUD**	**GTST-1-B**	
	**First Stage**	**Second Stage**
1	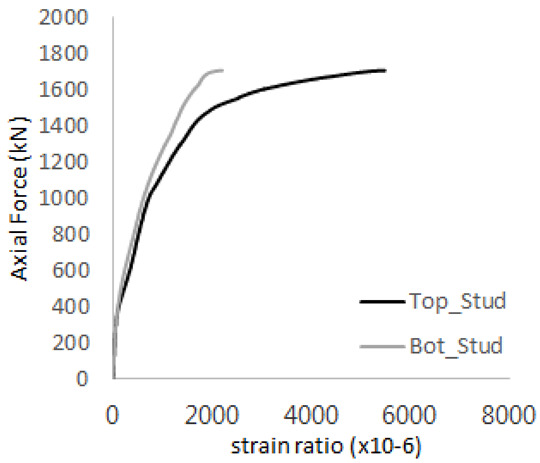	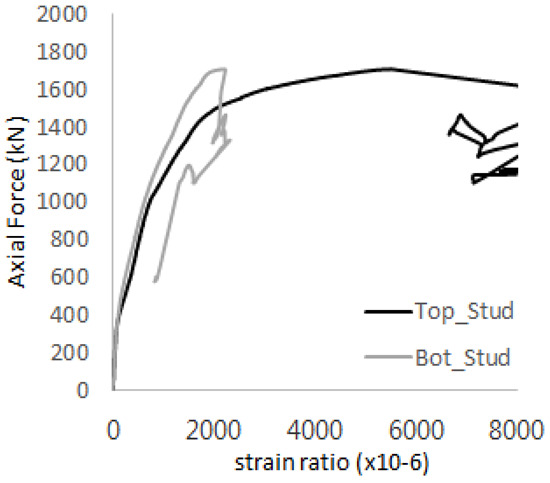
2	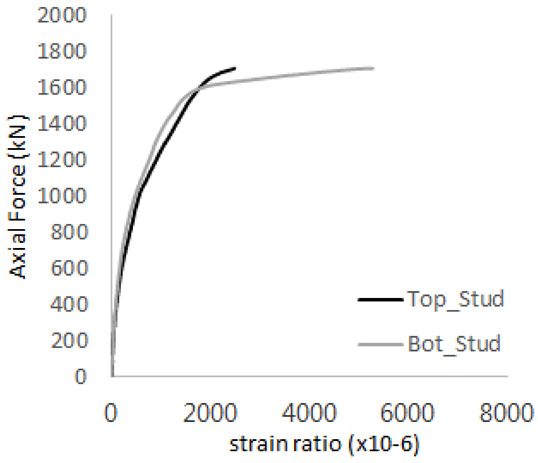	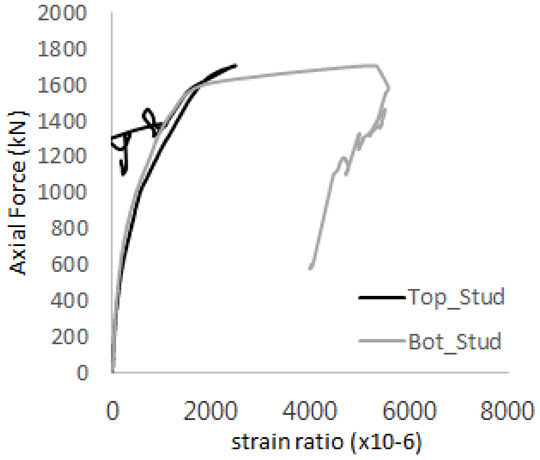
3	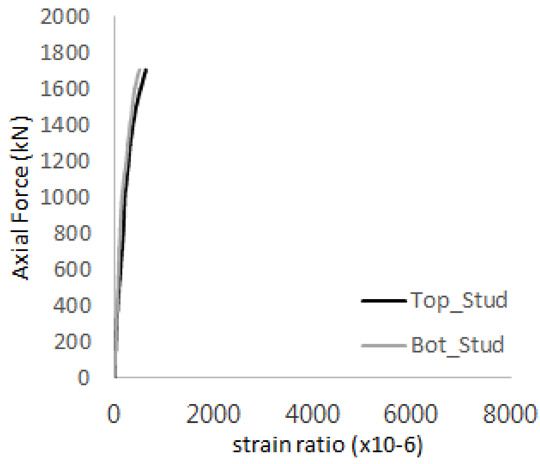	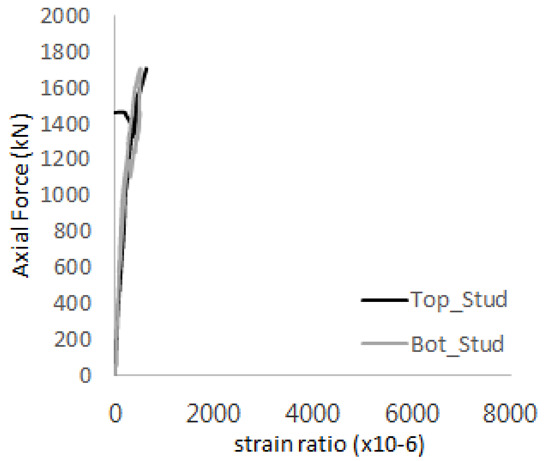
4	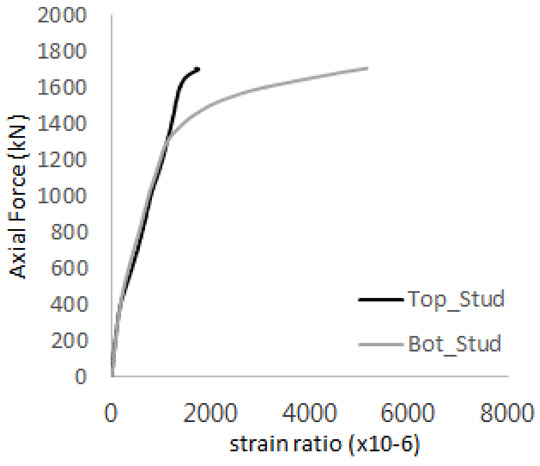	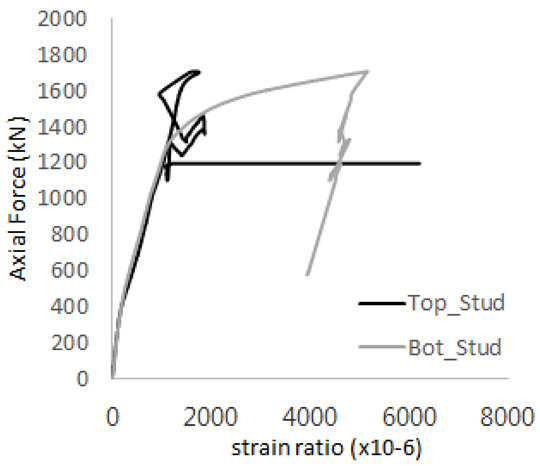
5	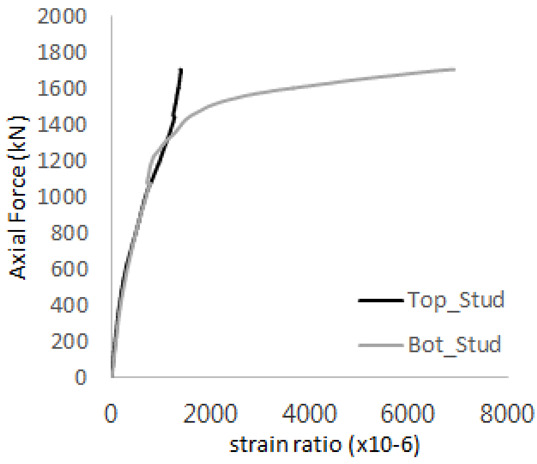	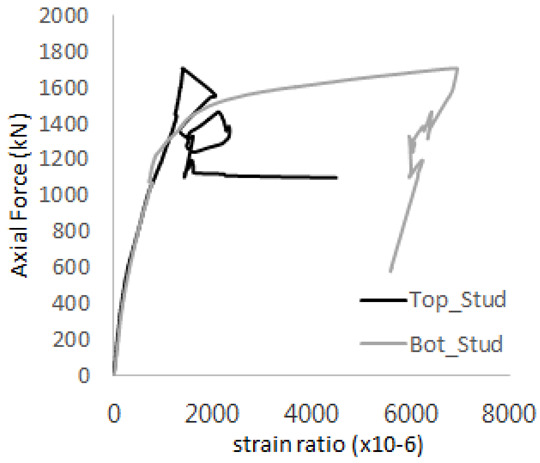

**Table 9:** Histories of strain stages for tie bars (strain ratio, GTST-1).

**Tie**	**GTST-1-A**	
	**First Stage**	**Second Stage**
1	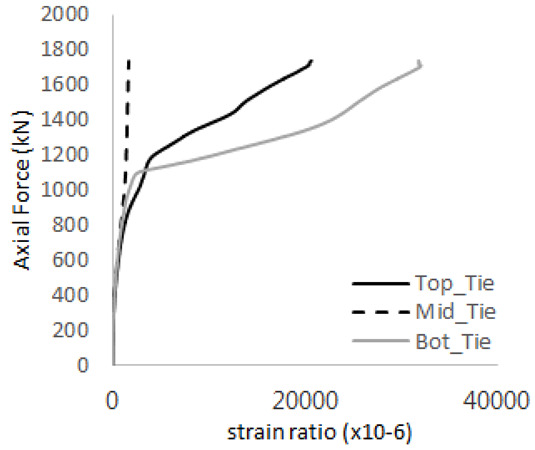	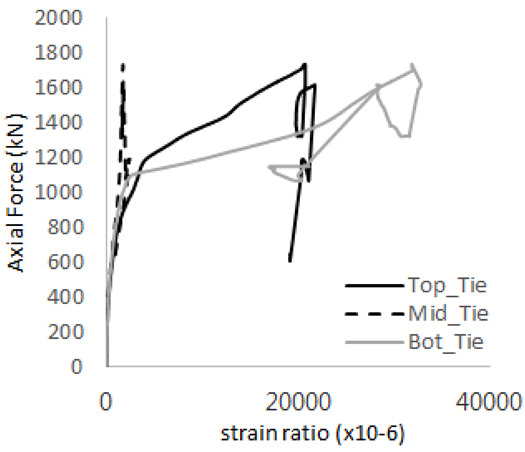
2	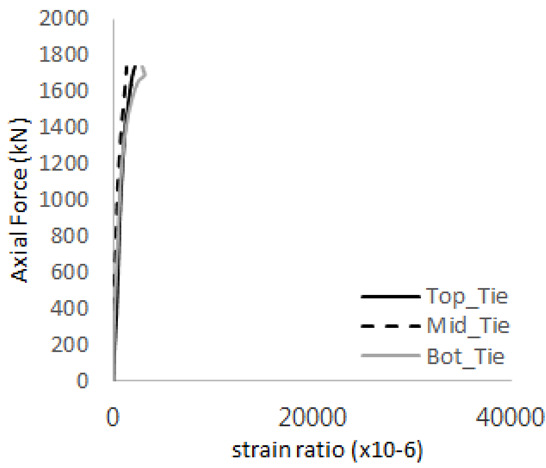	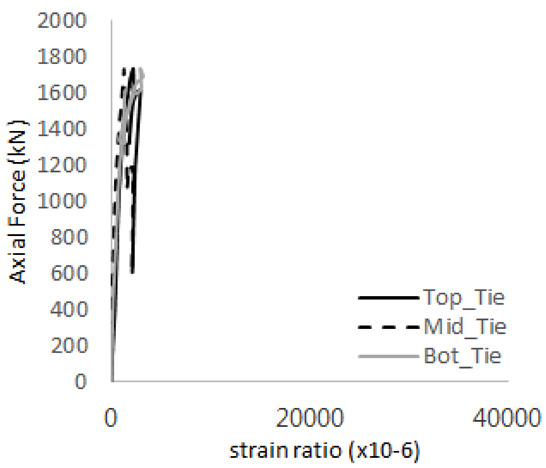
3	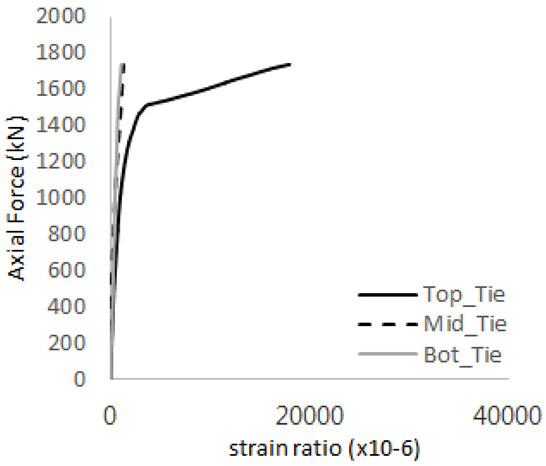	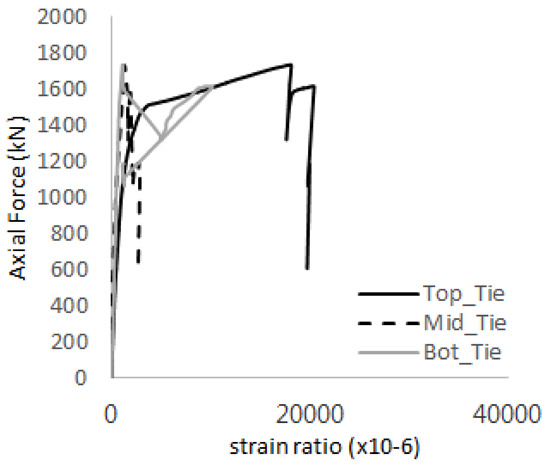
4	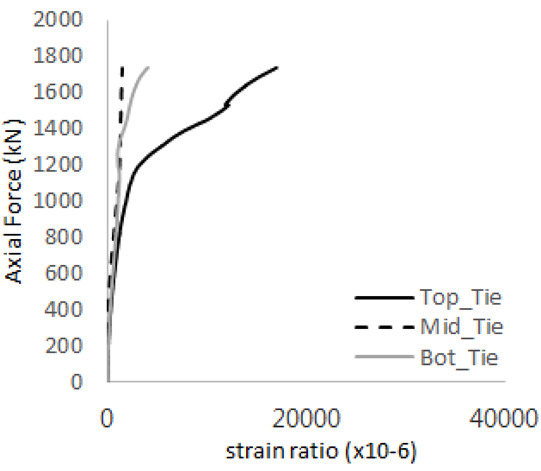	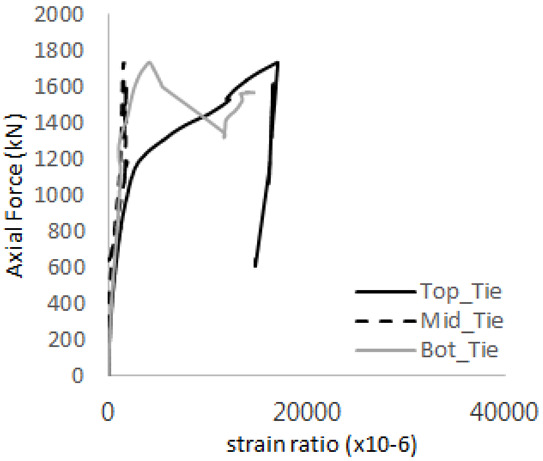
**Tie**	**GTST-1-B**	
	**First Stage**	**Second Stage**
1	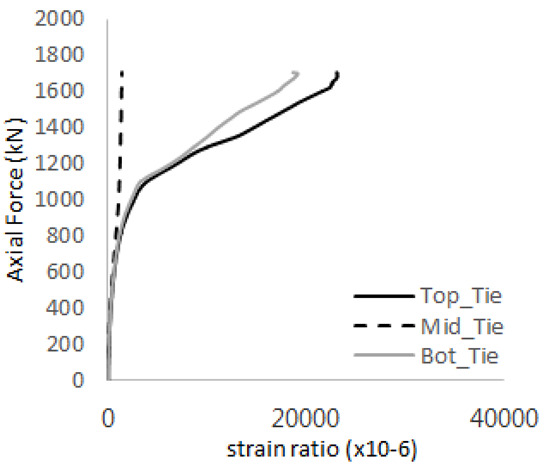	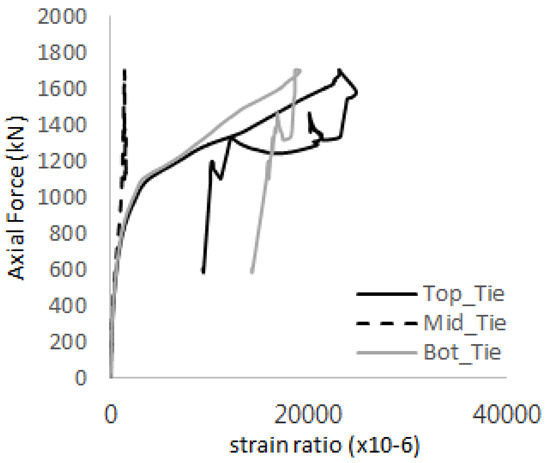
2	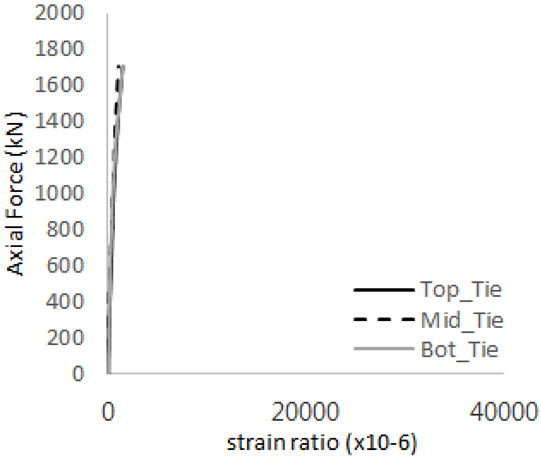	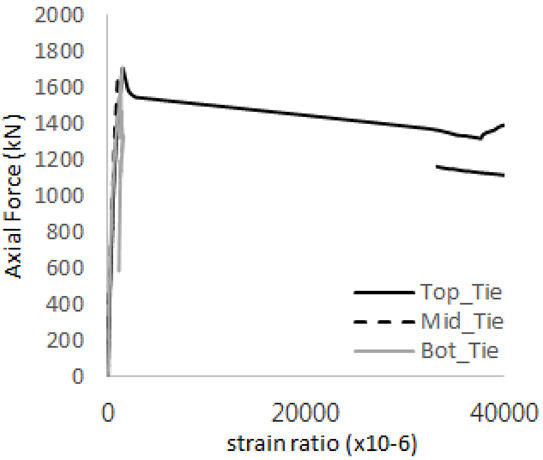
3	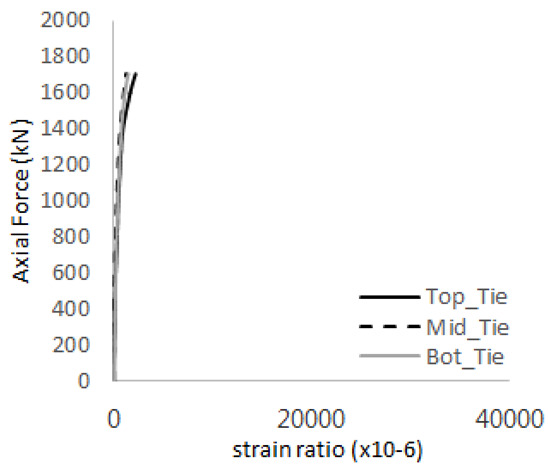	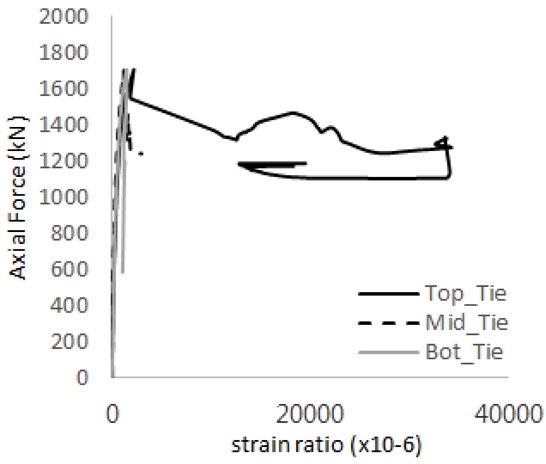
4	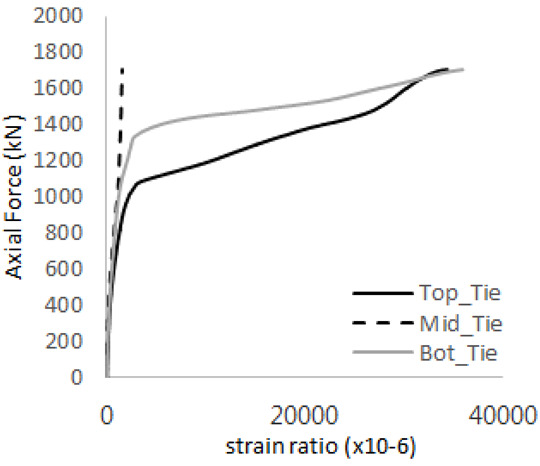	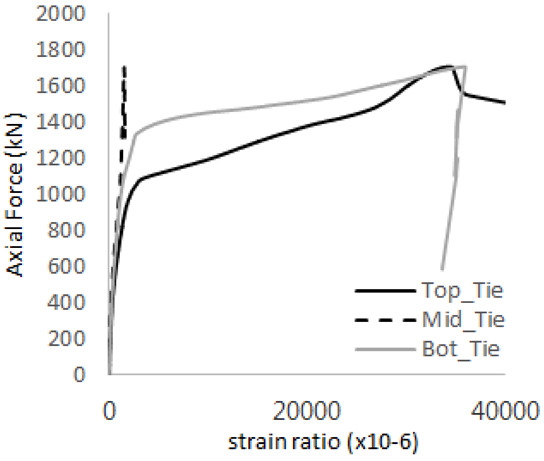

**Table 10:** Results comparison (tests/calculations).

Prediction	GTST-2-A	GTST-2-B
(a)	1.58	1.69
(b)	1.02	1.09
(c)	0.92	0.98
(d)	0.81	0.87

**Table 11:** Histories of strain stage for studs (strain ratio, GTST-2).

**STUD No.**	**GTST-2-A**	
	**First Stage**	**Second Stage**
1	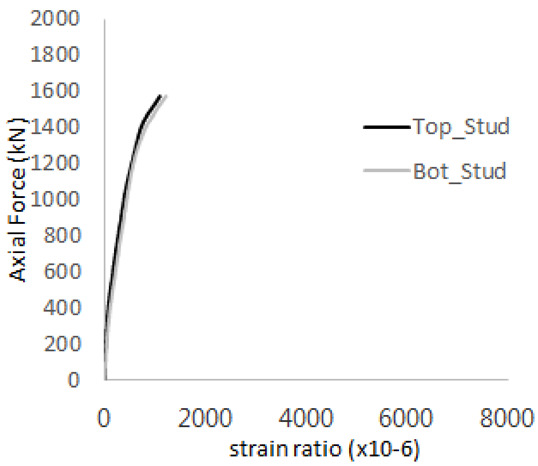	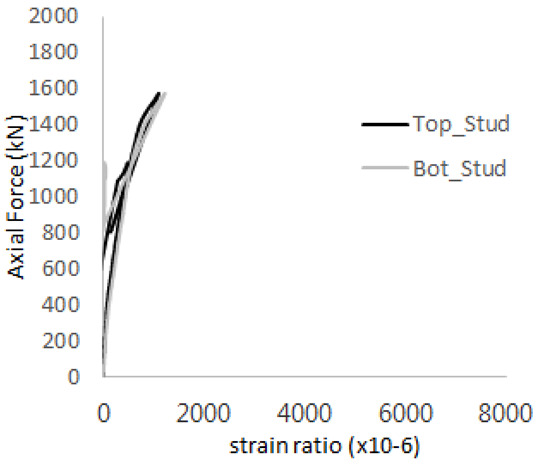
2	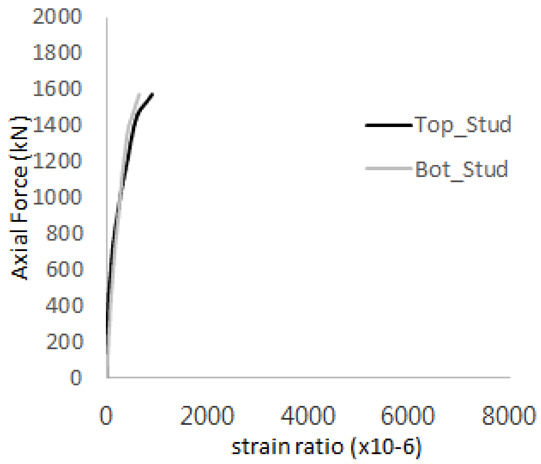	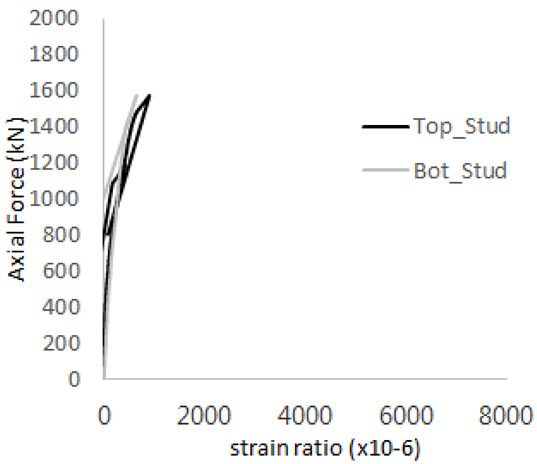
3	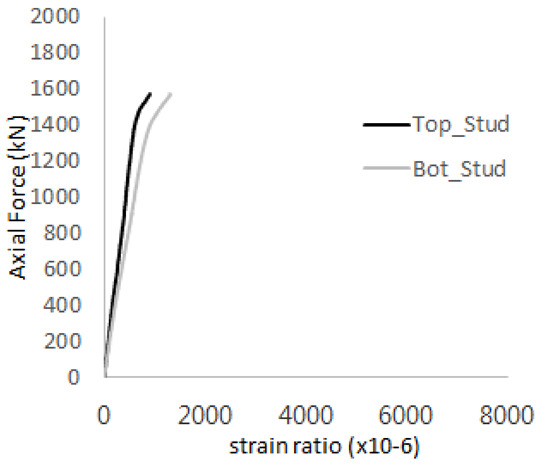	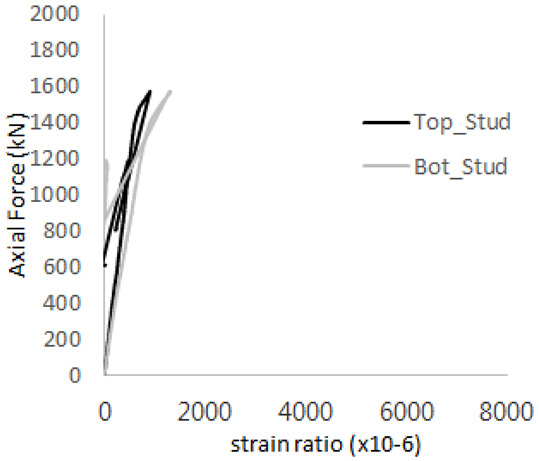
4	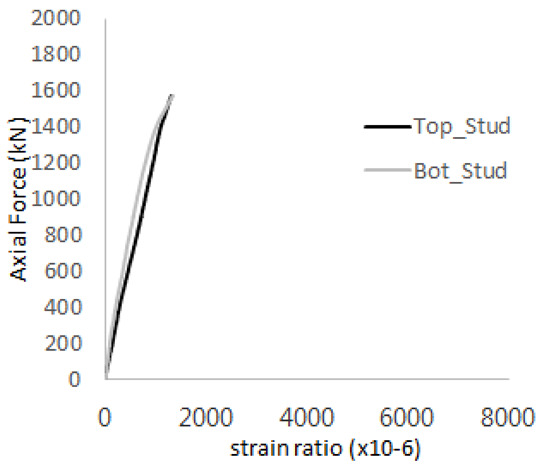	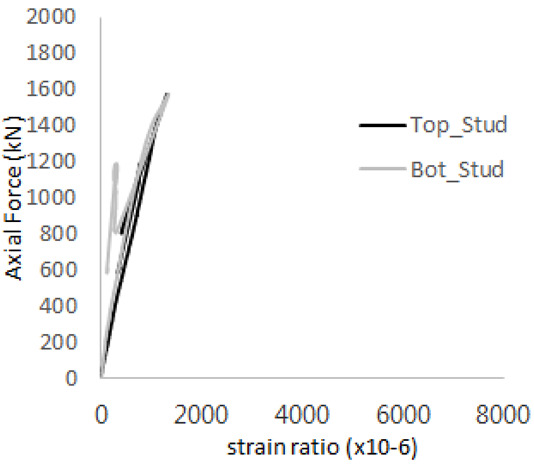
5	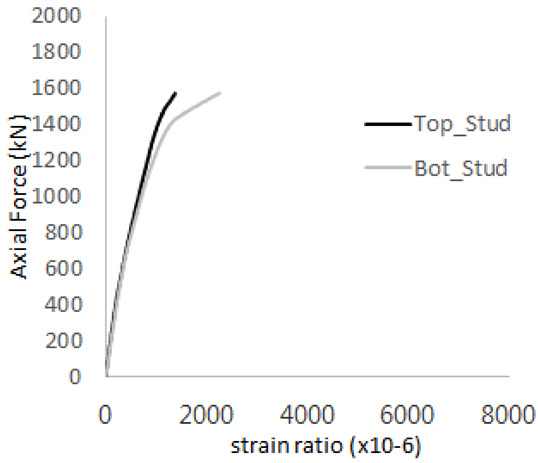	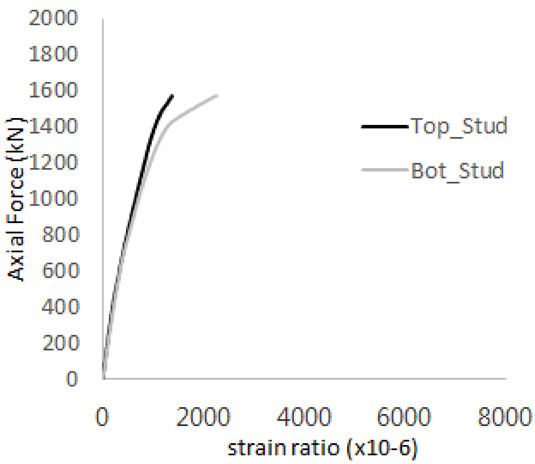
**STUD No.**	**GTST-2-B**	
	**First Stage**	**Second Stage**
1	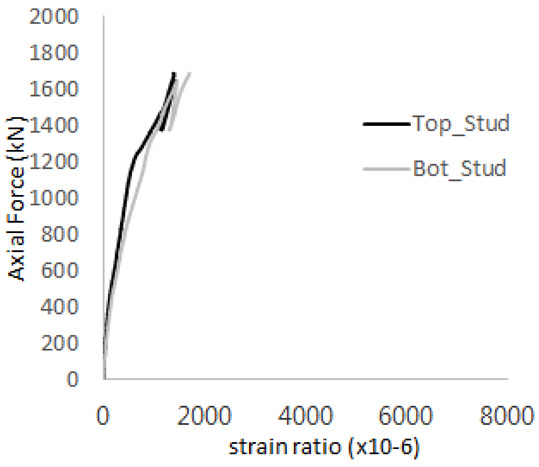	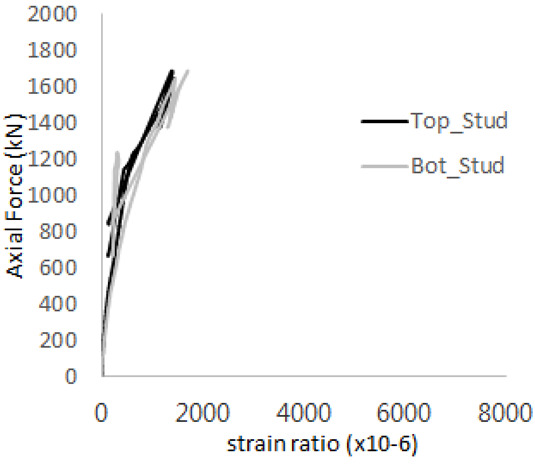
2	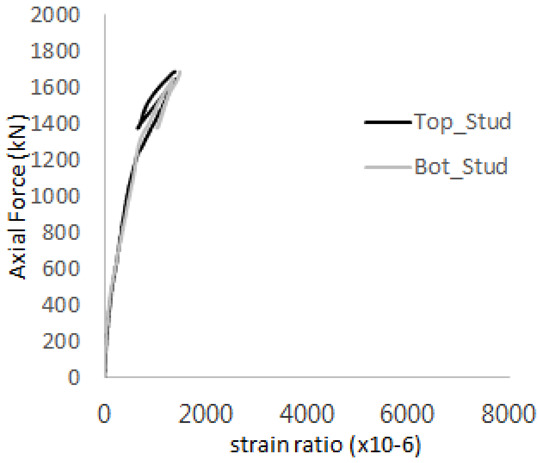	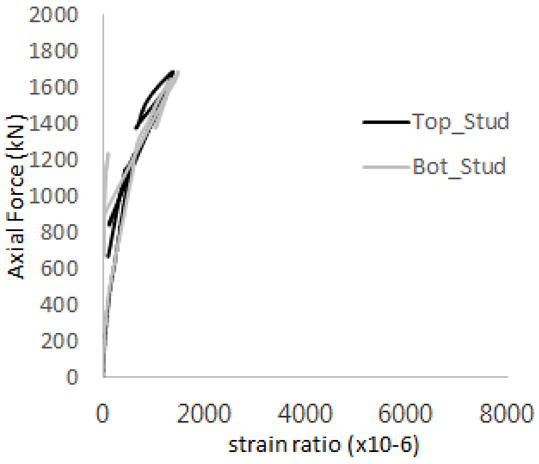
3	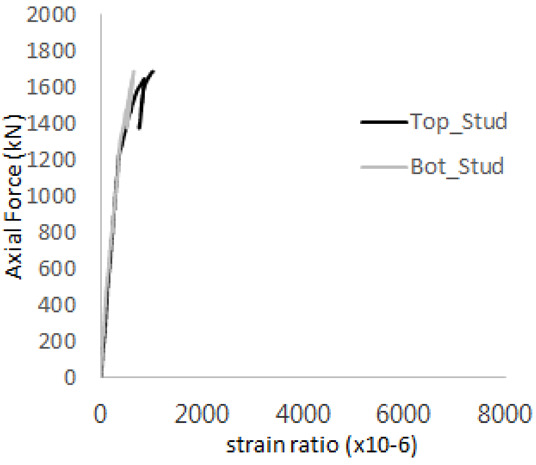	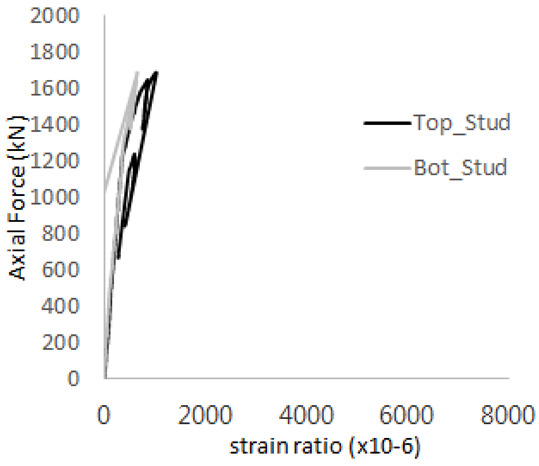
4	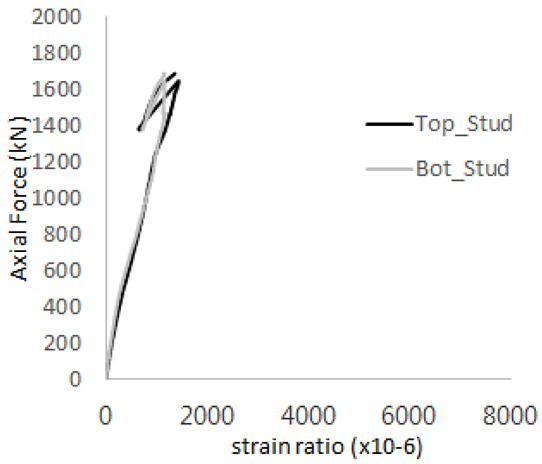	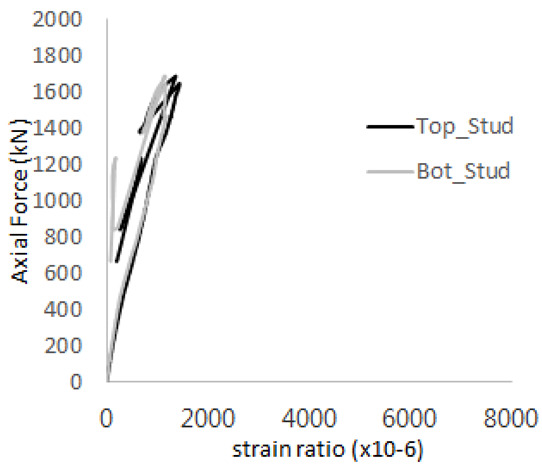
5	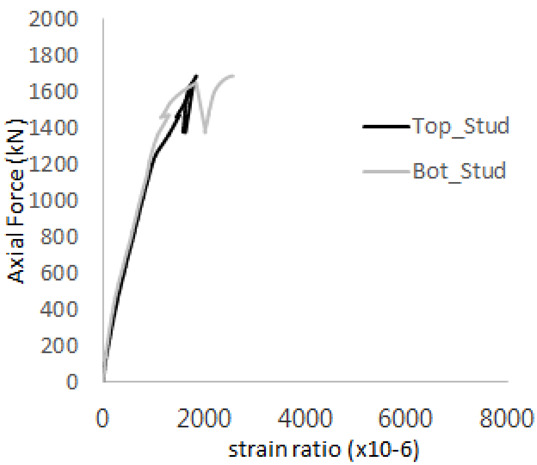	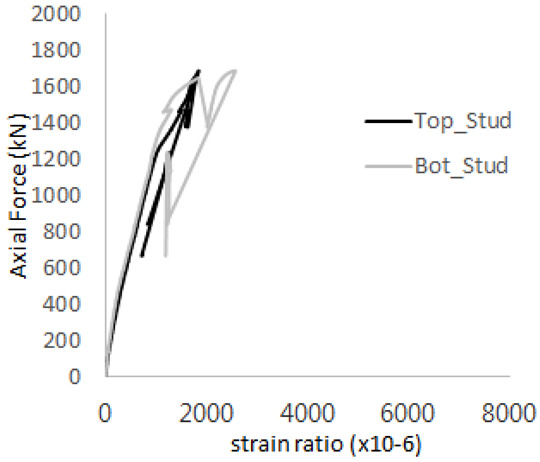

**Table 12:** Histories of tie bars (strain ratio, GTST-2).

**Tie No.**	**GTST-2-A**	
	**First Stage**	**Second Stage**
1	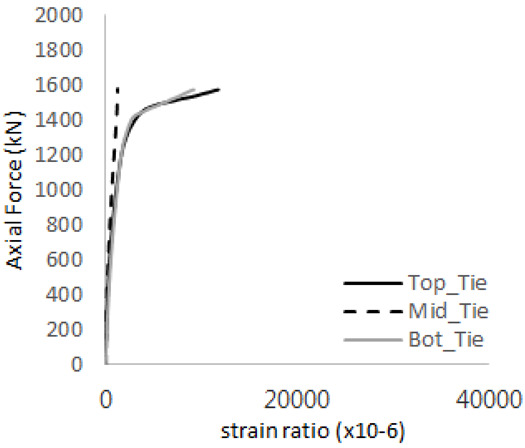	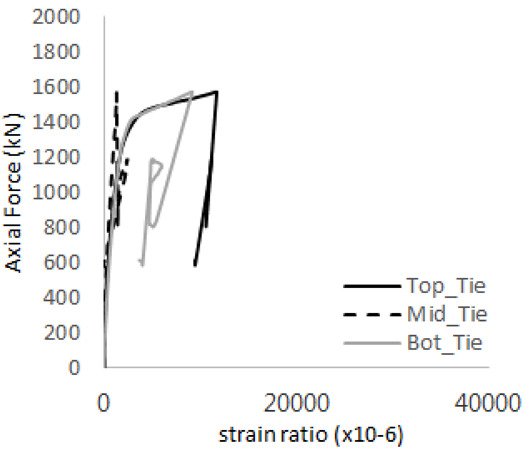
2	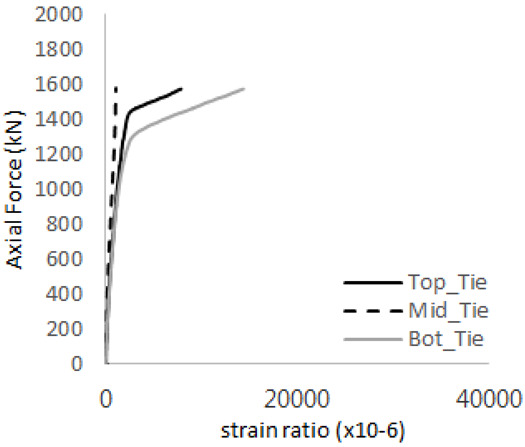	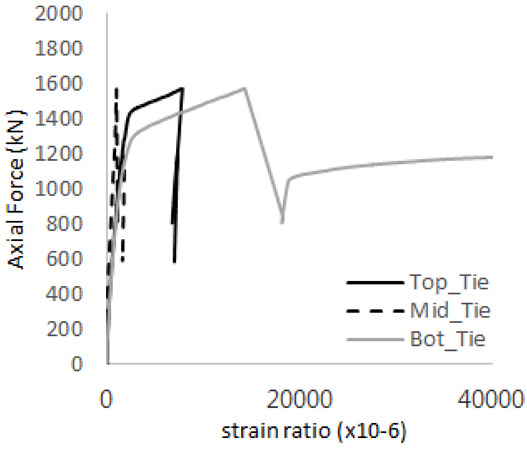
3	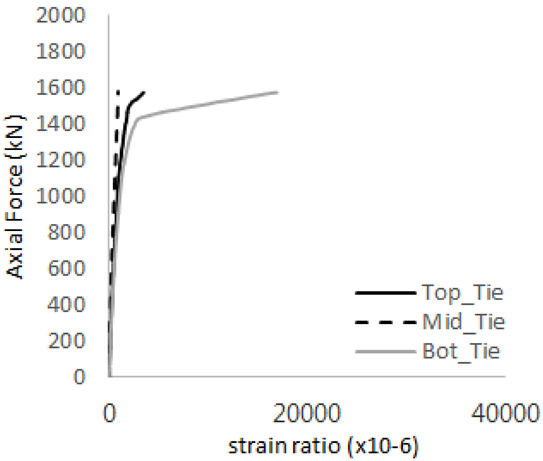	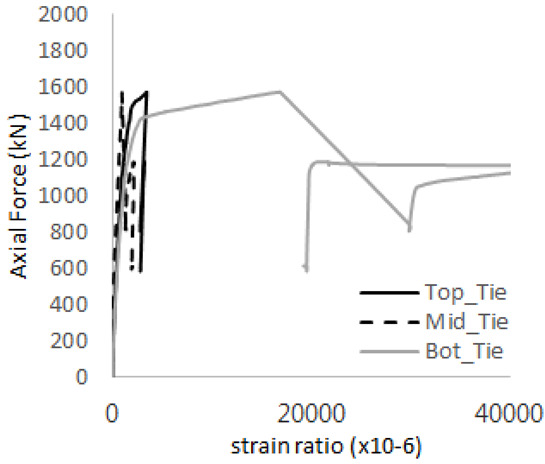
4	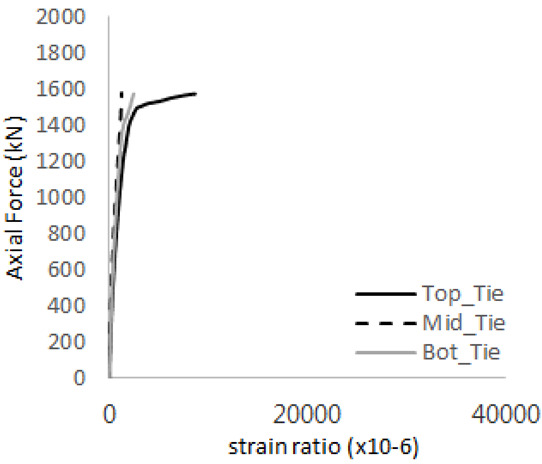	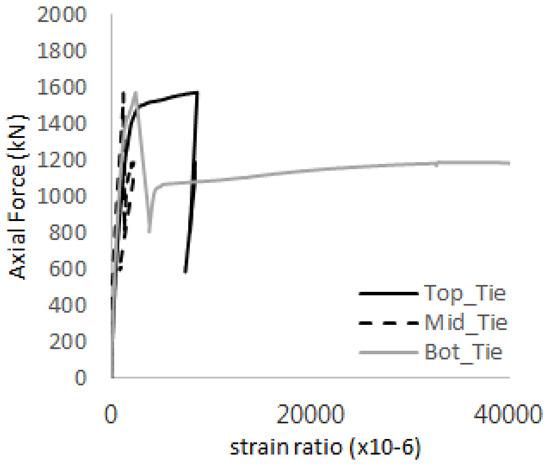
**Tie No.**	**GTST-2-B**	
	**First Stage**	**Second Stage**
1	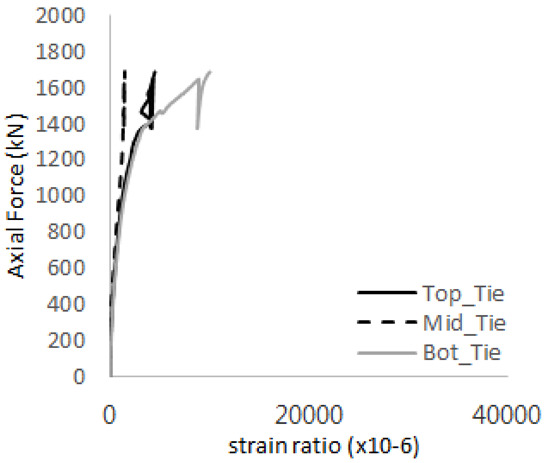	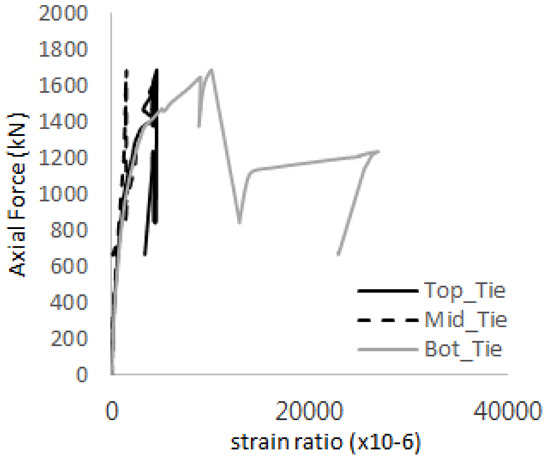
2	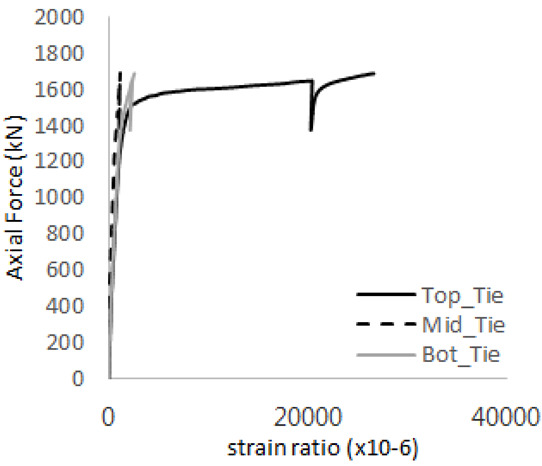	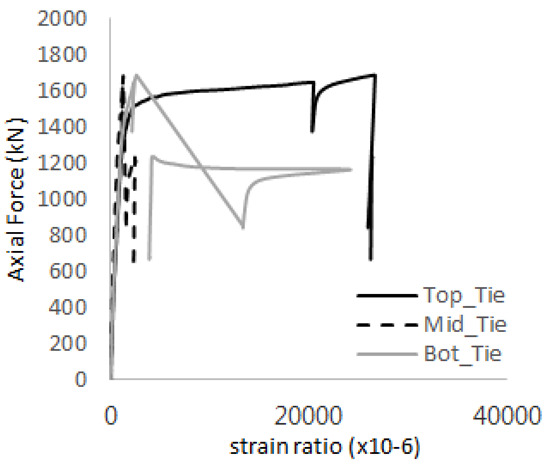
3	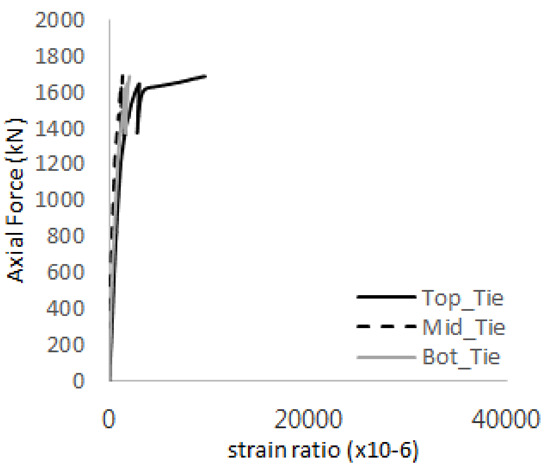	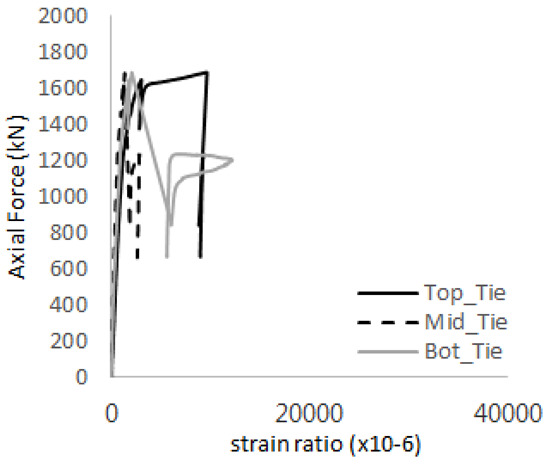
4	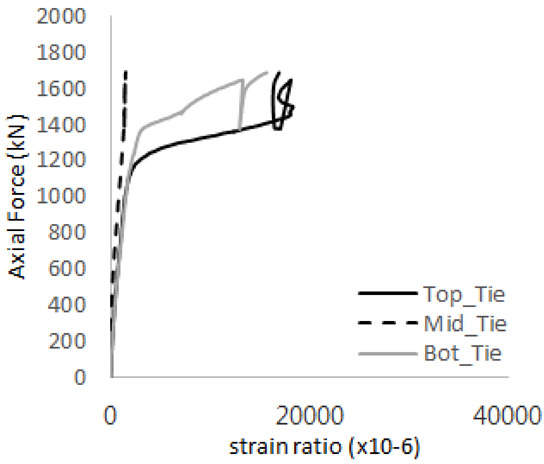	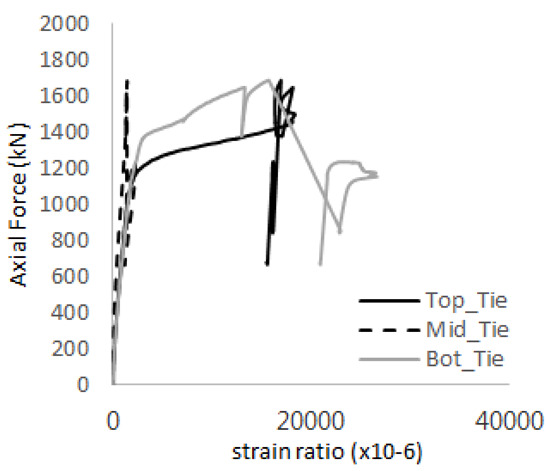

**Table 13:** Tensile strain length of each tie bar (mm).

Tie Bar No.	GTST-1-A			GTST-1-B			GTST-2-A			GTST-2-B		
	$\delta_{i}$	$l_{1}$	$l_{2}$	$\delta_{i}$	$l_{1}$	$l_{2}$	$\delta_{i}$	$l_{1}$	$l_{2}$	$\delta_{i}$	$l_{1}$	$l_{2}$
1	9.339	134.3	206.7	5.819	142.7	115.4	1.756	63.3	49.5	1.211	18.9	41.9
2	1.110	125.3	166.7	0.551	0	0	1.839	44.3	80.8	2.290	107.9	10.5
3	3.532	316.8	19.7	0.654	0	0	1.687	21.1	103.2	0.976	42.5	9.0
4	3.902	271.1	65.1	9.688	128.4	134.1	0.977	49.8	14.2	2.563	60.5	56.1
Avg.	4.471	211.9	114.5	4.178	67.8	62.4	1.565	44.6	61.9	1.760	57.4	29.4

**Table 14:** Effective plastic tie length of GTSTs.

Locations	GTST-1-A	GTST-1-B	GTST-2-A	GTST-2-B
Avg. top ($l_{1})$	8.47D (=211.9)	2.71D (=135.6)	1.79D (=44.6)	2.30D (=57.4)
Avg. bottom ($l_{2})$	4.58D (=114.5)	2.50D (=124.8)	2.48D (=61.9)	1.17D (=29.4)
Average	6.52D	2.60D	2.13D	1.74D
Overall average	≅4.56D		≅1.94D	

**Table 15:** Effective area of studs $A_{Nc,t}$ (GTST).

Effective Length		GTST-1s	GTST-2s
2D * (=50 mm)	$A_{Nc,t}$	720,000 mm^2^	457,000 mm^2^
	$n_{eff,s}$	4.73	3.01 EA **
3D (=75 mm)	$A_{Nc,t}$	650,700 mm^2^	358,800 mm^2^
	$n_{eff,s}$	4.28 EA	2.36 EA
5D (=125 mm)	$A_{Nc,t}$	411,000 mm^2^	186,500 mm^2^
	$n_{eff,s}$	2.70 EA	1.23 EA

* D = diameter of tie bar (25 mm); ** EA: number of effective studs to adopt modification effect.

**Table 16:** Results comparison ((test)/(Equations (10) and (11))).

Equations	GTST-1 (300 mm)						GTST-2 (200 mm)					
	GTST-1-A			GTST-1-B			GTST-2-A			GTST-2-B		
	2D	3D	5D	2D	3D	5D	2D	3D	5D	2D	3D	5D
Equation (10)	1.01	1.05	1.24	0.99	1.03	1.22	1.09	1.17	1.36	1.17	1.26	1.46
Equation (11)	0.99	1.03	1.22	0.97	1.01	1.20	0.99	1.08	1.29	1.06	1.16	1.38
Case (a)	1.53			1.50			1.58			1.69		
Case (b)	0.98			0.97			1.02			1.09		
Case (c)	0.96			0.94			0.92			0.98		

## Data Availability

The original contributions presented in the study are included in the article, further inquiries can be directed to the corresponding author.

## Nomenclatures

$A_{Nc}$	projected concrete failure area of anchors for calculation of strength in tension
$A_{Nc0}$	projected concrete failure area of a single anchor for calculation of strength in tension
$A_{se}$	effective cross-sectional area of anchor
$A_{brg}$	bearing area of headed stud or anchor bolt
$A_{tie}$	area of tie bar
D	diameter of tie or stud
$F_{u}$	nominal tensile stress
$F_{u,test}$	tensile stress by material test
$f_{c}^{\prime }$	nominal compressive cylinder stress of concrete at 28 days
$f_{uta}$	specified tensile strength of anchor steel
$f_{ya}$	specified yield strength of anchor steel
$h_{ef}$	effective height of stud for anchoring
$K_{c}$	coefficient for basic concrete breakout strength in tension
$N_{b}$	basic concrete breakout strength in tension of a single anchor in cracked concrete
$N_{cbg}$	nominal concrete breakout strength in tension of a group of anchors
$N_{sa}$	nominal strength of a single anchor or group of anchors in tension
$N_{p}$	pullout strength in tension of a single anchor in cracked concrete
$n_{s}$	number of stud
$n_{tie}$	the number of tie
$n_{y,tie}$	minimum yield strength of tie bar
t	thickness of steel plate
$\psi_{ec,N}$	factor used to modify the tensile strength of anchors based on eccentricity
$\psi_{ed,N}$	factor used to modify the tensile strength of anchors based on proximity to the edges of concrete members
$\psi_{c,N}$	factor used to modify the tensile strength of anchors based on the presence or absence of cracks in concrete
$\psi_{cp,N}$	factor used to modify the tensile strength of post-installed anchors intended for use in uncracked concrete without supplementary reinforcement
